# Microalgae-Enriched High-Moisture Meat Analogues: Improved Physicochemical, Functional, and Digestibility Properties

**DOI:** 10.3390/foods14162838

**Published:** 2025-08-16

**Authors:** Wanida Pan-utai, Thidarat Pantoa, Waraporn Prasert, Janya Sangkhiaw, Catleya Rojviriya, Chalermluck Phoovasawat, Hataichanok Kantrong

**Affiliations:** 1 Department of Applied Microbiology, Institute of Food Research and Product Development, Kasetsart University, Bangkok 10900, Thailand; 2 Department of Food Chemistry and Physics, Institute of Food Research and Product Development, Kasetsart University, Bangkok 10900, Thailand; ifrtrp@ku.ac.th; 3 Department of Food Processing and Preservation, Institute of Food Research and Product Development, Kasetsart University, Bangkok 10900, Thailand; ifrwrpp@ku.ac.th (W.P.); ifrhnk@ku.ac.th (H.K.); 4 Food Quality Assurance Services Center, Institute of Food Research and Product Development, Kasetsart University, Bangkok 10900, Thailand; ifrjys@ku.ac.th; 5 Synchrotron Light Research Institute, 111 University Avenue, Suranaree, Mueang, Nakhon Ratchasima 30000, Thailand; catleya@slri.or.th (C.R.); chalermluck@slri.or.th (C.P.)

**Keywords:** microalgae, high-moisture meat analogues, plant-based proteins, functional, digestibility, characterisation

## Abstract

This investigation examined the effects of microalgae supplementation on the physicochemical properties, nutritional profile, and digestibility parameters of high-moisture meat analogues (HMMAs). The sustainability and nutritional potential of incorporating three microalgae species—*Arthrospira platensis*, *Haematococcus pluvialis*, and *Nannochloropsis oculata*—into diets were investigated at inclusion levels of 0.5% and 1.5% (*w*/*w*). Colour metrics, compositional analysis, antioxidant capacity, textural characteristics, and in vitro protein digestibility were also assessed. The findings demonstrated enhancements in nutritional quality, particularly in protein content. Antioxidant capacity was significantly elevated in the 1.5% inclusion samples. Samples containing 1.5% *A. platensis* exhibited the highest chlorophyll concentrations at 19.91 mg/mg, while 1.5% *H. pluvialis* displayed carotenoid levels at 34.59 µg/mg. These improvements correlated with increased efficacy in ABTS and FRAP radical scavenging assays. Colourimetric analysis indicated that elevated microalgae concentrations contributed to darker hues; 1.5% *H. pluvialis* markedly increased redness (a-value, *p* < 0.05), with the visual profile similar to conventional meat. Supplementation with 1.5% *A. platensis* consistently decreased hardness and chewiness, likely attributable to enhanced porosity. Conversely, 1.5% *N. oculata* promoted a honeycomb-like microstructure, thereby augmenting cut resistance and hardness. The diminished rehydration capacity observed in 1.5% *H. pluvialis* was ascribed to smaller pore sizes, but maintained a higher oil-holding capacity relative to the control. All microalgae-infused HMMAs retained excellent in vitro protein digestibility. These results underscored the potential of microalgae—particularly 1.5% *A. platensis* for nutritional and textural enhancements, 1.5% *H. pluvialis* for improved visual and antioxidant properties, and 1.5% *N. oculata* for elevated phenolic and chlorophyll contents—in advancing sustainable, plant-based meat alternatives.

## 1. Introduction

Microalgae are now increasingly recognised for their potential as a sustainable source of nutrition and bioactive compounds, due to their high growth rates that enable substantial biomass production in a short time [1]. Microalgae are a significant source of essential nutrients, providing complete proteins, omega-3 and omega-6 fatty acids, vitamins, and minerals, making them ideal additives for dietary supplementation, particularly for vegetarians and vegans [2]. Microalgae are also a source of various bioactive molecules, including antioxidants, polysaccharides, lipids, peptides, carotenoids, phenolic compounds, and fatty acids [3]. These compounds have potential health applications due to their antioxidant, antimicrobial, anti-inflammatory, anticancer, antidiabetic, antihypertensive, antihyperlipidemic, and antiobesity effects [4,5]. Microalgae can produce various primary and secondary metabolites and serve as therapeutic agents for many health disorders, making them valuable in the cosmetic, food, and pharmaceutical industries [6,7]. Several microalgal strains are significant protein sources, with some species containing up to 70% protein dry weight and all 20 essential amino acids needed to meet human dietary requirements [8]. Protein from microalgae is regarded as a more sustainable alternative compared to conventional sources such as meat [9]. *Arthrospira* is a microalgal genus of filamentous cyanobacteria and an excellent source of protein, containing a diverse range of essential and non-essential amino acids as a beneficial dietary supplement for overall health [3,10,11]. The microalga *Haematococcus pluvialis* is recognised as a prolific source of carotenoids, particularly astaxanthin, which is renowned for its high antioxidant properties and associated health benefits [12,13]. *Nannochloropsis* microalgae contain bioactive compounds that can be used as environmentally sustainable nutritional supplements for the intake of PUFAs and EPA, with the potential to generate functional food products [14]. Lipids and antioxidants enhance the nutritional value of meat analogues while also improving emulsification and oxidative stability during extrusion [15]. Despite this potential, *N. oculata* has received relatively limited attention in fibrous, extruded food matrices. This study explored the comparative effects of *N. oculata*, *A. platensis*, and *H. pluvialis* on the physicochemical, structural, and nutritional performance of high-moisture meat analogues (HMMAs). As the global population expands, environmental issues related to animal agriculture increase, with a critical need to develop sustainable and nutritionally sufficient alternatives to meat [16]. The production of conventional meat is linked to substantial greenhouse gas emissions, high consumption of land and water resources, and ethical dilemmas concerning animal welfare [17]. In response to these challenges, plant-based meat analogues have emerged as a viable solution [18]. Among the various categories of plant-based meat analogues, HMMAs, primarily produced using high-moisture extrusion (HME) technology, show considerable potential due to their capability to mimic the fibrous texture and juiciness characteristic of meat from animals [19,20]. However, traditional HMMAs, generally made from soy, wheat, or pea proteins often fall short in nutritional adequacy, functional properties, and digestibility [21], underscoring the necessity to investigate novel, nutrient-rich ingredients that can enhance product quality while adhering to sustainability principles. Soy protein isolate (SPI) and wheat gluten (WG) are commonly used as base proteins in the formulation of HMMAs due to their high protein content and favourable texturising properties [19]. SPI and WG are essential in HMMAs due to their complementary properties. SPI, with over 90% protein, offers excellent emulsification and water retention and a neutral flavour. WG, rich in glutenin and gliadin, develops a fibrous texture through disulphide bonding during high-moisture extrusion [22,23]. Defatted soy flour (DFSF), containing 38–50% protein and residual fibre, serves as a cost-effective protein source that improves water retention and matrix cohesion without significantly increasing costs [24], ensuring that meat analogues effectively mimic the texture and mouthfeel of traditional meat.

Microalgae have garnered significant attention as a sustainable, protein-rich food source due to their high nutrient content and functional properties. Several studies have explored the incorporation of microalgae into snacks, bakery products, and low-moisture protein matrices [25,26,27], but their application in HMMAs remains underexplored, especially in relation to their techno-functional contributions beyond pigmentation. Previous studies have focused on the colour-enhancing potential of *Haematococcus pluvialis* in HMMA formulations, highlighting its astaxanthin content and stability during processing and storage [28]. However, these studies largely emphasised visual or sensory attributes, with limited evaluation of how different microalgae affect the physicochemical, water-related, and nutritional properties of HMMAs—particularly textural integrity (TI), rehydration capacity (RHC), cooking yield, and in vitro protein digestibility. A previous study showed that some microalgae, such as *Auxenochlorella protothecoides*, possess poor structurability due to their low protein–protein interaction potential and altered electrolyte environment [29]. Previous studies demonstrated that incorporating microalgae into food products at levels below 2% enhanced their nutritional and functional properties without compromising texture or sensory quality [30]. However, increasing the microalgae concentration resulted in a darker colour and an undesirable appearance, which impacted consumer acceptance. HMMAs can mimic meat-like textures, but they frequently lack nutritional adequacy, functional efficacy, and digestibility. To overcome these challenges, components like legume proteins, dietary fibres, hydrocolloids, and plant-based lipids have been added to enhance texture, emulsification, and nutritional quality [19,31]. Integrating microalgae into HMMAs presents ongoing technological and compositional challenges [29,32]. The pigmentation and biochemical profiles of microalgae species significantly influence their physical attributes and extrusion performance [33]. Incorporating microalgae influences key quality parameters such as texture profile, water-holding capacity, thermal stability, and emulsification behaviour, depending on the species and formulation used [25,34]. In vitro digestibility assessments, including standard gastric and intestinal simulations, are crucial for elucidating nutrient bioaccessibility, protein degradation patterns, and antioxidant retention [32,35,36]. Such evaluations are vital for substantiating the nutritional advantages of microalgae-based HMMAs and guiding the development of sustainable functional foods [33].

In this study, three microalgae species—*Arthrospira platensis*, *Haematococcus pluvialis*, and *Nannochloropsis oculata*—were integrated into the HME process to improve the functional, nutritional, and digestibility characteristics of HMMAs. Our results contribute to the growing body of knowledge on sustainable food innovation and offer practical solutions for the formulation of next-generation plant-based meat alternatives.

## 2. Materials and Methods

### 2.1. Microalgae and Ingredients

Three microalgae species, *Arthrospira platensis*, *Haematococcus pluvialis*, and *Nannochloropsis oculata* were integrated into the HME process. The *A. platensis* IFRPD 1182 biomass was cultivated in Zarrouk medium [37]. The harvested cells were dried in a hot air oven at 65 °C for 6 h. After drying, the biomass was milled using a grinder to achieve a consistent particle size of 0.5 mm. The processed biomass was then packed in aluminium bags and stored in a dark environment until needed for the experiments. The microalgae species *H. pluvialis* and *N. oculata* were procured from T.S. Twin Product Company Limited, Thailand, and their biochemical compositions were analysed and presented in Table 1. Defatted soy flour (DFSF) and soy protein isolate (SPI) were sourced from Win, Chan Industries, Thailand, and wheat gluten (WG) was purchased from a local bakery.

### 2.2. Microalgae Integrated into High-Moisture Extrusion (HME) Experiments

Preliminary experiments with microalgae-based HMMAs revealed that incorporating 30% *A. platensis* led to excessive darkening and greening of the product, which adversely affected its visual appeal (Figure S1). Higher incorporation levels increased formulation costs, posing challenges for product scalability. Consequently, the selected levels strike a balance among nutritional enhancement, acceptable appearance, and cost-effectiveness.

The HME experiments were developed using a combination of soy protein and wheat, incorporating three species of microalgae (*A. platensis* (A), *H. pluvialis* (H), and *N. oculata* (N)) at concentrations of 0.5% (level 1) and 1.5% (level 2) (*w*/*w*), designated as A1 and A2 for *A. platensis*, H1 and H2 for *H. pluvialis*, and N1 and N2 for *N. oculata*. Microalgae supplementation was excluded from the control experiments. The mixture formulations included 49.50% dehydrated food source feed (DFSF), which contained 50.18% protein, 1.69% fat, 34.15% carbohydrate, and 21.50% fibre. The mixture also included 20% soy protein isolate (SPI) and 30% wheat gluten (WG). The microalgae were incorporated at 0.5% for the first level and 1.5% for the second level, with the DFSF content adjusted to 48.50% for the latter formulation. All the ingredients, including the microalgae biomass, were utilised in dry powder form, because the high-moisture extrusion process necessitates meticulous control of moisture and thermal inputs. Before extrusion, dry microalgae were manually blended with the other powdered components to achieve homogeneous mixing.

High-moisture extrusion (HME) was performed using an intermeshing, co-rotating twin-screw extruder (Clextral Evolum 25, Firminy, France) featuring a screw diameter of 25 mm and an L/D ratio of 24:1. The extrusion barrel comprised six temperature-controlled zones and was connected to a square-shaped, elongated cooling die to facilitate the development of a fibrous structure. The maximum temperature was maintained at 135 °C in the sixth barrel section. Water was continuously injected at a flow rate of 6.8 kg/h, while dry feed was supplied at a rate of 3.7 kg/h, with screw rotational speed maintained at 500 rpm throughout the process [38]. The screw configuration incorporated multiple conveying elements separated by kneading blocks, along with a single reverse element situated in the mid-barrel section to generate sufficient back pressure and shear forces necessary for protein alignment. The functions of the screw element type in each barrel zone section are listed in Supplementary Table S1. Following the extrusion process, samples were collected directly from the die and manually cut into uniform dimensions of 3.0 cm (width) × 6.0 cm (length) × 0.8 cm (height), with a measurement tolerance of ±2.0 mm. These dimensions represented the post-extrusion state before conditioning. Each treatment was conducted with two independent biological replicates, representing separate extrusion batches. Samples from each batch were analysed in triplicate for all measurements. The extrudate samples were stored in sealed laminated plastic bags at −20 °C and thawed at 4 °C for 12 h before use.

### 2.3. Physicochemical Properties

#### 2.3.1. Colour Measurements

Colour changes among the microalgae-incorporated HMMA samples and the control were measured using a Datacolour Spectraflash Spectrophotometer (SF 600 Plus; Datacolour International Co., Lawrenceville, NJ, USA). Colour measurements were reported in terms of lightness (L*), ranging from 0 (black) to 100 (white), and chromaticity parameters a*, spanning from green (−) to red (+) and b*, from blue (−) to yellow (+).

#### 2.3.2. pH, Moisture Content, and Water Activity

The HMMAs incorporating microalgae and the control samples were immersed in deionised water and subjected to homogenisation for 1 min. The pH of the mixture was determined using a pH meter (Schott Instruments, Lab850, Mainz, Germany). The moisture content was measured by drying the samples in an oven at 105 °C overnight or until a constant weight was achieved, with results expressed as a percentage. This moisture content, calculated on a wet basis, represented the proportion of water relative to the total weight of the sample. Water activity (a_w_) was measured using a resistive electrolytic humidity measuring system at 25 °C (LabMaster-aw, Novasina AG, Lachen, Switzerland).

#### 2.3.3. Textural Integrity

The textural integrity (TI) assessment was conducted by rehydrating a 5 g portion of the torn sample in 100 mL of boiling water for 30 min. The sample was then drained in a colander for 10 min, ground with a meat grinder, rinsed under running tap water for 1 min, and drained again before drying in a hot air oven at 105 °C for 3 h. The dried weight of the samples was recorded, and the textural integrity was calculated using the following equation [39]:

(1) $$TI\left(\%\right)=\frac{\left(W_{I}-W_{F}\right)}{W_{I}}\times 100$$

where $W_{I}$ and $W_{F}$ denote the initial and final weights of the sample, respectively.

### 2.4. Textural Properties

The textural properties were analysed following a previous report [40]. Two methodologies were utilised to assess the textural characteristics of the sample. Texture profile analysis (TPA) was conducted using a Texture Analyzer (Model TA-XT2i, Stable Micro Systems Ltd., Godalming, UK) equipped with a p/25 cylinder probe. The HMMAs incorporating microalgae and the control sample, each measuring 3 cm in length and 3 cm in width, were analysed at a pre-test speed of 1 mm/s, a test speed of 1 mm/s, and a post-test speed of 1 mm/s. The samples were then compressed to 60% of their original height and subjected to double compression testing to evaluate their textural properties. A blade set with a Warner Bratzler: HDP/BSW probe was applied to measure hardness. Samples measuring 1.5 cm in length and 1.5 cm in width were penetrated by the probe at a speed of 2.0 mm/s, corresponding to 30% of their height.

### 2.5. Nutritional Properties

The proximate compositions of the microalgae biomass and the HMMAs incorporating microalgae with the control sample were analysed following AOAC standard methods [41]. The crude protein content was determined through micro-Kjeldahl methods. Crude lipids were extracted using Soxhlet extraction with petroleum ether and then dried to a constant weight. The crude fibre content was assessed for acid and alkaline digestion, with the resulting fibre residue dried to a constant weight. The ash content was evaluated by igniting the dried samples in an electric furnace at 550 °C, with the carbohydrate content calculated by subtracting the sum of moisture, protein, lipid, fibre, and ash from 100 g of dry matter.

### 2.6. Pigment Determination

The HMMAs incorporating microalgae and the control sample were extracted using methanol at a ratio of 1:5 g/mL. The mixture was thoroughly mixed and then stored in the dark at 4 °C for 24 h. The extractant was separated by centrifugation at 5000 rpm for 10 min. The optical density of the supernatant was measured at 662, 645, and 450 nm using a spectrophotometer (Model SP-8001, UV/Vis, Metertech Inc., Taipei, Taiwan), with methanol serving as the blank. The total chlorophyll and carotenoid contents were calculated using the following equations [42,43]:

(2) $$Chlorophyll\;a\left(mg/mL\right)={11.75\times OD}_{662}-2.350\times {OD}_{645}.$$

(3) $$Chlorophyll\;b\left(mg/mL\right)={18.61\times OD}_{645}-3.960\times {OD}_{662}.$$

(4) $$Total\;carotenoids\left(\mu g/mL\right)=\frac{{[1000\times OD}_{470}-2.27\times Chlorophyll\;a-81.4\times Chlorophyll\;b]}{227}$$

The total chlorophyll content comprising the combined concentrations of chlorophyll a and b and the carotenoid concentration was expressed as milligrams per milligram of sample (mg/mg) and micrograms per milligram of sample (µg/mg), respectively.

### 2.7. Antioxidant Properties

The supernatant obtained from the preceding pigment determination was utilised to analyse the total phenolic content (TPC) and evaluate the antioxidant properties using the ABTS and FRAP assays.

The TPC of the extracted samples was assessed using the Folin–Ciocâlteu colourimetric method, with minor modifications [44]. In brief, 20 μL of the sample was combined with 100 µL of 0.2 N Folin–Ciocâlteu solution (SRL, Mumbai, India) and 80 µL of 0.7 M sodium carbonate solution, followed by incubation at room temperature for 8 min. Subsequently, 50 μL of distilled water was added to the mixture and incubated at 40 °C for 30 min. The absorbance was recorded at 750 nm using a microplate reader (M965+, Microplate Reader, Metertech, Taiwan). Gallic acid served as the standard. The results were expressed as mg gallic acid equivalent (mg GAE/g).

The antioxidant activity of the samples, indicated by scavenging ABTS radicals, was evaluated using a previous method, with minor adjustments [45]. In summary, the ABTS radical solution was created by reacting 505.05 µL of 7 mM ABTS (2,2-azino-bis (3-ethylbenzothiazoline-6-sulphonic acid) diammonium salt) (SRL, Mumbai, India) with 5.05 µL of 245 mM ammonium persulphate. This mixture was stored in the dark at room temperature for 16 h and then diluted with distilled water until reaching an optical density of 0.7 at 750 nm. Ten microlitres of the sample were then combined with 190 µL of the ABTS solution, and the combination was kept in the dark for 5 min. The absorbance was recorded at 750 nm using a microplate reader. Ascorbic acid (Sigma-Aldrich, Singapore) served as the standard antioxidant. The antioxidant capacity of the sample was expressed in mg of ascorbic acid equivalent (mg AAE/g).

A ferric ion reducing antioxidant power assay with the extracted samples from sequential phycobiliprotein extraction was performed according to the method of Renugadevi et al. [46], with slight modifications. The reagent was prepared from 300 mM sodium acetate (pH 3.6) and 10 mM TPTZ (2,4,6-tris (2-pyridyl)-*s*-triazine) (SRL, India) in 40 mM HCl and 20 mM ferric chloride (Sigma-Aldrich, Singapore) at volumes of 25, 2.5, and 2.5 mL, respectively. Then, a 10 μL sample was mixed with 190 µL of FRAP reagent before incubating in the dark for 30 min. The absorbance was measured at 593 nm using a microplate reader. Ascorbic acid (Sigma-Aldrich, Singapore) was used as the standard. All samples were determined in duplicate, with results expressed as mg ascorbic acid equivalent (mg AAE/g).

### 2.8. Techno-Functional Properties

#### 2.8.1. Rehydration Capacity

The rehydration capacity (RHC) of the samples was assessed using the method outlined by Charlie et al. (2025) [40]. Five grams of the samples were rehydrated in 100 mL of boiled deionised water for 30 min, drained for 10 min, and then weighed. The RHC was calculated using the equation

(5) $$RHC\left(\%\right)=\frac{\left(W_{2}-W_{1}\right)}{W_{1}}\times 100$$

where $W_{1}$ and $W_{2}$ are the weights of the sample before and after the rehydration process.

#### 2.8.2. Cooking Yield

The cooking yield of the samples was assessed in accordance with the methodology established in a previous report [47]. Five grams of torn samples were placed in a mesh bag, subjected to hot water at 80 °C for 20 min, and then allowed to drain for 10 min before weighing. The cooking yield was calculated using Equation (6).

(6) $$Cooking\;yield\left(\%\right)=\frac{W_{2}}{W_{1}}\times 100$$

where $W_{1}$ and $W_{2}$ are the weights of the sample before and after boiling.

#### 2.8.3. Water-Holding Capacity

The water-holding capacity (WHC) was assessed employing a modified methodology based on previous approaches [40]. The samples were dried using a freeze-dryer (Labconco, Kansas City, MO, USA). The freeze-dried samples were then ground into a fine powder. One gram of the powdered sample ${(W}_{0})$ was mixed with 10 mL of distilled water in a pre-weighed centrifuge tube ${(W}_{1})$ and vortexed for 10 s, followed by 5 min rest intervals, for a total duration of 30 min at 25 °C. The tube was then centrifuged at 3000× *g* for 30 min. After carefully discarding the supernatant, the remaining sample in the tube was weighed ${(W}_{2})$. The WHC was expressed as a percentage of grams of water per gram of sample as:

(7) $$WHC\left(\%\right)=\frac{{((W}_{2}{-W}_{1})-W_{0})}{W_{0}}\times 100$$

#### 2.8.4. Oil-Holding Capacity

The oil-holding capacity (OHC) was assessed using the method outlined in a previous report [40]. In each experiment, the freeze-dried samples ${(W}_{0})$ were combined with 10 mL of rice bran oil in a pre-weighed centrifuge tube ${(W}_{1})$. The mixture was vortexed for 10 s at 5 min intervals for a total duration of 30 min. The samples were then centrifuged at 3000× *g* for 30 min, and the supernatant was carefully removed. Any excess oil was eliminated by inverting the centrifuge tube before reweighing ${(W}_{2})$. The OHC was calculated as grams of oil absorbed per gram of dry sample using Equation (8).

(8) $$OHC\left(\%\right)=\frac{{((W}_{2}{-W}_{1})-W_{0})}{W_{0}}\times 100$$

### 2.9. Characterisation of Extruded Microalgae-Integrated High-Moisture Meat Analogues

#### 2.9.1. Scanning Electron Microscopy

The microstructure was examined by scanning electron microscopy (SEM) using a HITACHI SEM (Model SU3500, Hitachi High-Technologies Corporation, Tokyo, Japan). The SEM specimens were prepared by tearing, cutting, and rehydrating them and affixing them onto SEM stubs. All the samples were observed under the microscope at a magnification of ×200.

#### 2.9.2. Synchrotron Radiation X-Ray Tomographic Microscopy

A porosity analysis was conducted to examine the structure using synchrotron radiation X-ray tomography microscopy (SRXTM). The SRXTM experiments were performed on all freeze-dried HMMA samples using a filtered polychromatic X-ray beam, characterised by a mean energy of 11.5 keV, at the XTM Beamline (BL1.2 W) of the 1.2 GeV Siam Photon Source facility. Each sample was positioned on an air-bearing rotary stage within the experimental station for X-ray tomography data collection. The X-ray projections were gathered at an isotropic voxel size of 3.61 µm utilising an imaging system that included a 100-micron thick YAG:Ce scintillator (CRYTUR, Turnov, Czech Republic), a 2× objective lens-coupled microscope (Optique Peter, Lentilly, France), and a Neo 5.5 camera (Andor Technology Ltd., Belfast, Northern Ireland, UK). During a typical tomography scan, X-ray projections were collected over a 180-degree rotation, with an angular increment of 0.30 degrees. The tomographic volume of each sample was reconstructed from enlarged composite projections obtained through multiple scans (ranging from 6 to 10, depending on the sample size). The first scan covered 180°, followed by additional 180° scans that were systematically shifted along the vertical axis of rotation. The image processing of all X-ray projections included flat-field correction, cropping, spot filtering, and beam intensity normalisation before tomographic reconstruction using Octopus Reconstruction software version 8.9.1 (TESCAN, Gent, Belgium). The tomographic volumes were subsequently visualised and presented in 3D graphics using Drishti software version 2.6.4 [48].

### 2.10. Amino Acid Determination

The amino acid compositions of the microalgae-incorporated HMMA samples at the maximum concentration of microalgae (level 2) and the control sample were analysed in accordance with a previously established method, with minor modifications [49]. The samples underwent hydrolysis using 6 M HCl and 4 M NaOH (specifically for tryptophan) in a Teflon screw-capped glass tube, maintained on a heating block at 110 °C for 24 h. After hydrolysis, the pH of the hydrolysate was adjusted to 2.2 in an ice bath using 10 M NaOH. The samples were subsequently filtered through a 0.45 μm syringe filter membrane. Analysis was performed utilising a high-performance liquid chromatography (HPLC) device (Agilent HP1260, Agilent Technologies, Waldbronn, Germany) equipped with a fluorescence detector (FLD). This process included pre-column derivatisation with o-phthaldialdehyde (OPA). The fluorescence of the OPA derivatives was monitored at excitation and emission wavelengths of 340 nm and 450 nm, respectively. For OPA derivatisation, 10 μL of the sample was mixed with 2.5 μL of OPA and allowed to react for 5 min before injection into the column. Derivatisation of proline was performed using 0.5% FMOC, with 500 μL of borate buffer mixed with 150 μL of the sample and 125 μL of FMOC. This mixture was vortexed for 30 s until a clear solution formed before placing it in a vial and loading it onto the autosampler. Chromatography was performed using an AdvanceBio AAA column (4.6 × 100 mm, 5 μm), incubated at 35 °C with a flow rate of 0.7 mL/min and an injection volume of 15 μL. The gradient mobile phase consisted of NaH_2_PO_4_ (pH 7.8), as well as a mixture of methanol, acetonitrile, and deionised water in a 45:45:10 ratio, with a total run time of 33 min. An amino acid analytical standard (AAS18, Merck, Burlington, MA, USA) was utilised for calibration.

### 2.11. In Vitro Digestion

The protein digestibility of microalgae-incorporated HMMA samples was evaluated at the maximum concentration of microalgae (level 2) for A2, H2, and N2 and compared with the control experiment, followed by characterisation using SDS-PAGE [50,51].

Oral digestion

The simulated salivary fluid (SSF) consisted of lysozyme (6 μg/mL), human salivary amylase (29.7 U/g carbohydrate), and urea (3 mM) in 0.15 M NaCl. The pH of SSF was adjusted to 6.9. The samples (100 mg) were vortexed with 372 μL of distilled water before adding pre-warmed simulated salivary fluid (SSF) at 37 °C (118 μL) and vortexing for 30 s. The enzyme reaction in orally digested or chewed (C) samples was stopped by placing them in an ice bath for 10 min before storing at −20 °C.

Gastric digestion

The simulated gastric fluid (SGF) consisted of 0.9 mM NaH_2_PO_4_, 3 mM CaCl_2_, 0.1 M HCl, 0.15 M NaCl, and 16 mM KCl dissolved in distilled water pH 2.5. SGF (118 μL) containing 63 U pepsin/mg protein was added to the orally digested sample. The mixture was shaken in a water bath (37 °C) for 0.3, 11, 22, 33, 44, 55, 66, 77, or 120 min. Enzymatic reactions were stopped by increasing the pH to 7.5 using 0.5 M NaHCO_3_ before storing at −20 °C.

Duodenal digestion

The hepatic mix solution (HMS), consisting of 12.5 mM sodium taurocholate, 12.5 mM sodium glycol-deoxycholate, 146 mM NaCl, 2.6 mM CaCl_2_, 4.8 mM KCl, and 4 mM cholesterol, was dissolved in distilled water. The pancreatic mix solution (PMS) consisted of 0.6 mM CaCl_2_, 4.1 μM ZnSO_4_, 125 mM NaCl, and 0.3 mM MgCl_2_ in distilled water. The PMS contained the intestinal enzymes 34.5 U/mg trypsin/mg protein, 11.8 U/mg protein of α-chymotrypsin, and 1.7 U/mg protein of pancreatic amylase. Each sample with enzyme was incubated in a shaking water bath (37 °C) at 170 rpm for 0.3, 5, 15, 30, 60, or 120 min. The enzymatic reaction was stopped using 0.1 M phenyl methyl sulphonyl fluoride (20 μL). The samples were placed in an ice bath for 10 min after each time point of the digestion phase, centrifuged (14,000× *g*), and separated into liquid (soluble) and solid (insoluble) samples. Protein digestibility and protein profiles after digestion were assessed using SDS-PAGE.

SDS-PAGE

The digested samples were characterised using SDS-PAGE according to Pantoa et al. (2020) [50]. The pellet (solid phase) was weighed (10 ± 1 mg) and extracted with 500 mL of 50 mM Tris-HCl buffer containing 50 mM dithiothreitol (DTT), 7 M urea, 2 M thiourea, and 2% CHAPS (3-[(3-Cholamidopropyl) dimethylammonio]-1-propane sulphonate), pH 8.8. The solution was sonicated at 60 °C for 5 min (repeated three times) and then centrifuged (14,000× *g*) at 4 °C for 30 min. The supernatant (50 μL) was mixed with 50 μL of Laemmli sample buffer (Bio-Rad) containing 5% of 2-mercaptoethanol (Bio-Rad Laboratories, Hercules, CA, USA) before heating to 100 °C for 5 min. Samples and 10–200 kDa protein markers (Precision Plus Protein All Blue Standards™, Bio-Rad Laboratories) were loaded onto a 12% acrylamide separating gel. Electrophoresis was performed at 200 V for 40 min. The gel was fixed in methanol (50%, *v*/*v*) and 10% (*v*/*v*) trichloroacetic acid (Sigma-Aldrich, St. Louis, MO, USA) for 2 h, then washed three times with distilled water and stained with Coomassie brilliant blue R-250 for 15 min before destaining overnight with continuous shaking.

### 2.12. Statistical Analysis

The results were presented as mean values ± standard deviation (SD) for duplicate biological experiments, with statistical analyses performed using SPSS version 25.0 (IBM Corp., Armonk, NY, USA). All the experimental parameters were analysed by one-way ANOVA and compared using Duncan’s multiple range test (DMRT) at a significance level of 0.05.

## 3. Results

Three species of microalgae, *A. platensis*, *H. pluvialis*, and *N. oculata*, were incorporated into a high-moisture extrusion (HME) process, and an evaluation of high-moisture extruded plant-based meat products incorporating microalgae was conducted to assess their physicochemical, textural, nutritional, biological, and techno-functional properties. Characterisation and digestibility studies were also performed to further analyse their potential applications.

**Figure 1:**
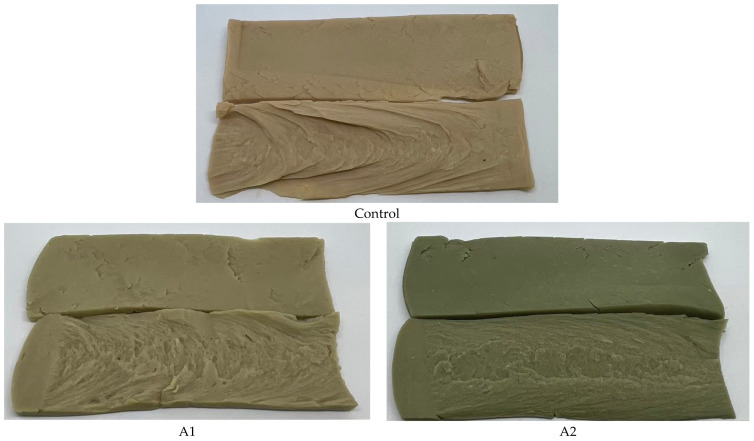

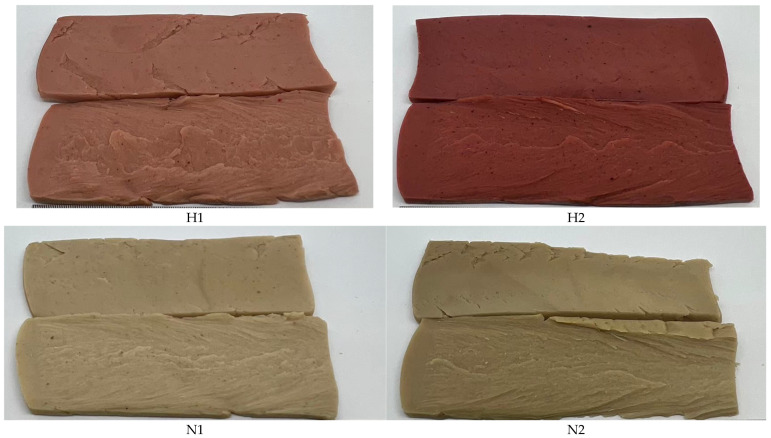
The appearance of high-moisture extruded plant-based meat incorporating 0.5% and 1.5% concentrations of microalgae: *A. platensis* (**A1**,**A2**), *H. pluvialis* (**H1**,**H2**) and *N. oculata* (**N1**,**N2**), without microalgae (**control**).

Figure 1 shows the texture and colour of the three microalgal strains incorporated into the HME process at 0.5% and 1.5% concentrations. The microalgae-supplemented products were similar those produced without microalgae, except for noticeable differences in colour. Higher concentrations of microalgae resulted in darker shades of green, red, and yellow from *A. platensis*, *H. pluvialis*, and *N. oculata* respectively.

### 3.1. Physicochemical

The experimental results (Table 2) revealed that the lightness (L*) values ranged from 43.76 to 74.21 and were significantly different from the control, with the exception of N1. Higher concentrations (1.5%) of each microalgal strain were associated with darker colour values. The highest green to red (a*) values were recorded in H1 and H2 and increased at higher concentrations of *H. pluvialis*. The b* value, representing the chromaticity from blue to yellow, indicated an increase in yellowness, specifically for *N. oculata* treatments N1 and N2. However, this increase was not statistically significant when compared to the control sample. All the colour values exhibited a similar trend to the naked eye, indicating high moisture content across the various conditions.

Table 3 presents the pH values, moisture content, water activity (a_w_), and textural integrity from a series of experiments focused on the HME process. The pH values ranged from 4.68 to 6.65, indicating a consistent level of acidity and alkalinity across the various experimental conditions. This suggested that the chemical stability of the samples was maintained throughout the testing period. Moisture content varied between 55.51% and 67.51% (wet basis), while water activity (a_w_) was between 0.89 and 0.90, highlighting the capability of the samples to effectively retain moisture. Both these parameters are critical determinants of the quality and shelf life of the extruded products.

The textural integrity demonstrated the ability of the samples to maintain a fibrous structure, resist fragmentation, and deliver the expected sensory characteristics of chewiness and bite—attributes that are essential for consumer acceptance of plant-based meat alternatives. Strong textural coherence is vital for ensuring that meat substitutes closely mimic the experience of consuming actual meat, which is crucial for their success in the market. The N1 and N2 experiments exhibited the highest levels of textural integrity. The significant differences between the experimental groups and the control underscored the impact of specific formulations on the quality and consumer appeal of plant-based meat substitutes.

### 3.2. Textural

Table 4 presents the textural properties of high-moisture, plant-based extruded meat analogues incorporated with microalgae. An increase in microalgae content during HME led to higher cutting force values. The addition of microalgae did not significantly influence the cutting force for formulas derived from *A. platensis* and *N. oculata* but impacted the cutting force for the two formulas incorporating *H. pluvialis*. The addition of microalgae reduced the hardness values compared to the control, with the exception of H1 and N2, which did not significantly differ from the control. A similar trend was found in chewiness, with microalgae generally decreasing chewiness values. Formula A1 did not exhibit a significant difference in chewiness compared to the control, while N2 showed an increase in the chewiness value. This variation was attributed to the inherent specific properties of each type of microalgae.

### 3.3. Nutritional

Three species of microalgae biomass were employed as significant ingredients in the HME process, with their biochemical compositions presented in Table 1. Each microalgae species had a different biochemical profile, reflecting its unique biological characteristics. *A. platensis* contained the highest levels of protein and fibre, which are essential for nutritional applications. By contrast, *N. oculata* exhibited the richest lipid content, making it a valuable source of healthy fats. *H. pluvialis* “set itself apart” with the highest carbohydrate content, indicating its potential for energy-dense formulations. These variations in biochemical compositions highlighted the potential to select microalgae species based on specific nutritional requirements and industrial applications.

The nutritional profiles of high-moisture plant-based meat analogues enhanced by the incorporation of microalgae are presented in Table 5. The protein contents ranged between 58% and 62% and were statistically insignificant across the different experimental conditions, highlighting the uniformity of the product’s nutritional attributes. The HMMAs base formulation consisted of 49.5% dehydrated food source feed (DFSF; ~50% protein), 20% soy protein isolate (SPI; ~90% protein), and 30% wheat gluten (WG; ~75% protein). This formulation was designed to yield a total protein content of 60%, consistent with the values obtained from protein analysis of the final extrudates. Microalgae, supplemented at low levels (0.5% and 1.5%), had a negligible impact on the overall protein content. The levels of ash and carbohydrate also remained consistent, with no significant differences observed between the experiments. This consistency was attributed to the low concentration of microalgae utilised in the HME process, which resulted in only minor variations in the nutritional compositions of the plant-based meat analogues.

### 3.4. Pigments and Antioxidant

Microalgae serve as a valuable source of pigments that possess strong antioxidant properties [52]. The results in Table 6 show the content of the chlorophyll and carotenoids pigments for high-moisture extruded plant-based meat analogues incorporating microalgae. Increasing the levels of *A. platensis* and *N. oculata* significantly elevated chlorophyll levels. Conversely, higher concentrations of *H. pluvialis* led to increases in carotenoid content, enhancing the nutritional profile and colour of the products. The highest chlorophyll and carotenoid contents were associated with *A. platensis* and *H. pluvialis*, reaching impressive maximums of 19.91 mg/mg for chlorophyll in sample A2 and 34.59 µg/mg for carotenoids in sample H2. These results highlighted the potential of certain microalgae to improve the bioactive compound content and aesthetic appeal of plant-based meat alternatives.

The evaluation of TPC and antioxidant properties using the ABTS and FRAP methods is summarised in Table 7. The TPC ranged from 1.09 to 2.86 mg GAE/g, with the highest value observed in samples containing 0.5% *N. oculata* incorporated into HMMAs. Antioxidant capacity varied between 0.46 and 0.89 mg AAE/g for the ABTS method and 0.48 to 0.98 mg AAE/g for the FRAP method. The highest antioxidant values were recorded for HMMAs incorporating *H. pluvialis* via the ABTS and FRAP assays. The antioxidant activities of the three microalgae tested were greater than the control, with experiments involving microalgae-integrated HMMAs yielding products with enhanced pigment and antioxidant properties.

### 3.5. Techno-Functional 

The techno-functional attributes of products derived from microalgae are influenced by the presence of various nutrients and bioactive compounds, including proteins, carbohydrates, lipids, and other natural substances. The interactions among these components play a vital role in determining the properties of these products. The rehydration capacity and cooking yield results are detailed in Table 8. Rehydration capacity (RHC) measures the ability of the dried analogue to absorb water and regain its functional properties. Unlike TI, which evaluates structural robustness under moisture, RHC reflects the efficiency of water uptake—a key factor for product stability, shelf-life, and usability in rehydrated forms. Unlike the texture index (TI), RHC does not evaluate mechanical properties, emphasising water uptake behaviour. Cooking yield refers to the proportion of mass retained after cooking, indicating the retention of water and fat during thermal processing. A higher cooking yield signifies reduced moisture loss, with enhanced juiciness and improved product quality. For *A. platensis* (A1 and A2), increasing the quantity did not have a significant effect; however, when compared to the control formula, there was improvement in all the measured values. By contrast, *H. pluvialis* (H1 and H2) analyses revealed no significant differences in parameters compared to the control sample. For *N. oculata*, the rehydration capacity was not significantly impacted by increasing the amount of microalgae, but inclusion enhanced textural integrity and cooking yield.

A microalgal concentration of 1.5% in HMMAs significantly enhanced the overall properties, encompassing aspects from appearance to functional characteristics, compared to 0.5%. The experimental conditions with the highest microalgae levels (A2, H2, and N2) were compared to the control group, focusing on critical metrics such as water and oil-holding capacity, characterisation, and microstructural analysis. An in-depth evaluation of amino acid profiles and protein digestibility was also conducted.

**Figure 2:**
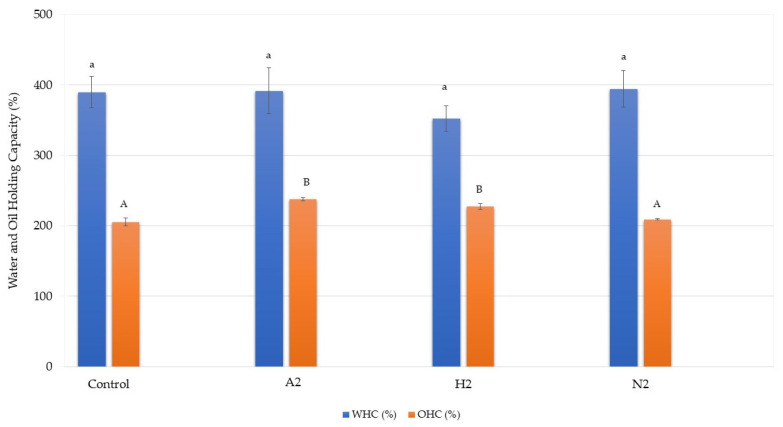
Water-holding capacity and oil-holding capacity of high-moisture extruded plant-based meat analogues incorporating microalgae. Data within the same parameter with different superscripts are significantly different (*p* < 0.05).

The water-holding capacity (WHC) and oil-holding capacity (OHC) results are illustrated in Figure 2. No statistically significant differences in WHC values were recorded across the three microalgal strains. The H2 experimental group exhibited the lowest WHC value, while samples treated with A2 and H2 demonstrated significantly higher OHC than the control and N2 groups. The variations in WHC and OHC were attributed to the distinct physiological and biochemical characteristics of each type of microalga utilised in the experiments. These differences underscored the importance of understanding the specific properties of microalgal strains when evaluating their potential applications in bioprocessing and bioengineering.

### 3.6. Characterisation

**Figure 3:**
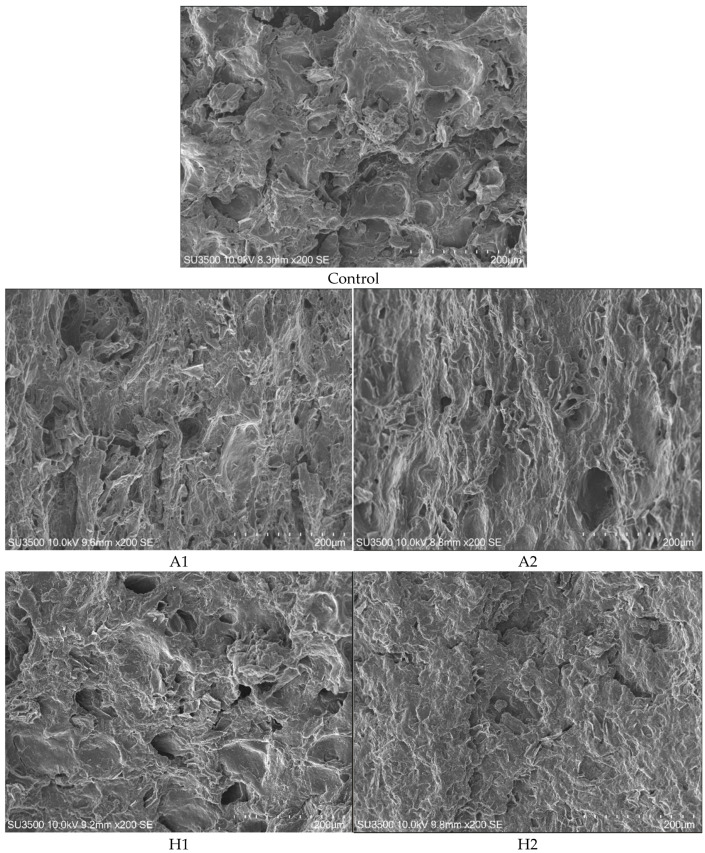

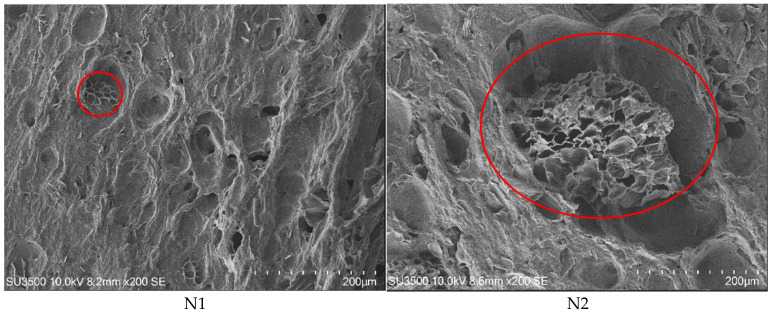
The microstructure of high-moisture extruded plant-based meat analogues incorporating microalgae under various conditions (magnification 200×). *A. platensis* (A), *H. pluvialis* (H), and *N. oculata* (N)) at concentrations of 0.5% (level 1) and 1.5% (level 2) (*w*/*w*), designated as (**A1**,**A2**) for *A. platensis*, (**H1**,**H2**) for *H. pluvialis*, and (**N1**,**N2**) for *N. oculata*. Microalgae supplementation was excluded from the (**control**) experiments.

The microstructural characteristics of HMMAs incorporating microalgae are depicted in Figure 3. A comparative analysis of the structural properties indicated that the samples incorporating *A. platensis* exhibited greater porosity than the control formulation. This increased porosity correlated with the enhanced rehydration capacity and cooking yield, leading to reduced hardness and chewiness. By contrast, the samples enriched with *H. pluvialis* showcased surface roughness, with H2 showing lower hardness and chewiness metrics compared to H1. Sample N2 had a distinctive microstructure that resembled a honeycomb arranged in a circular configuration. This unique architectural design contributed to a higher cutting force and increased the hardness and chewiness compared to sample N1. These findings highlighted how microstructural variations impacted the textural and physical properties of the samples.

**Figure 4:**
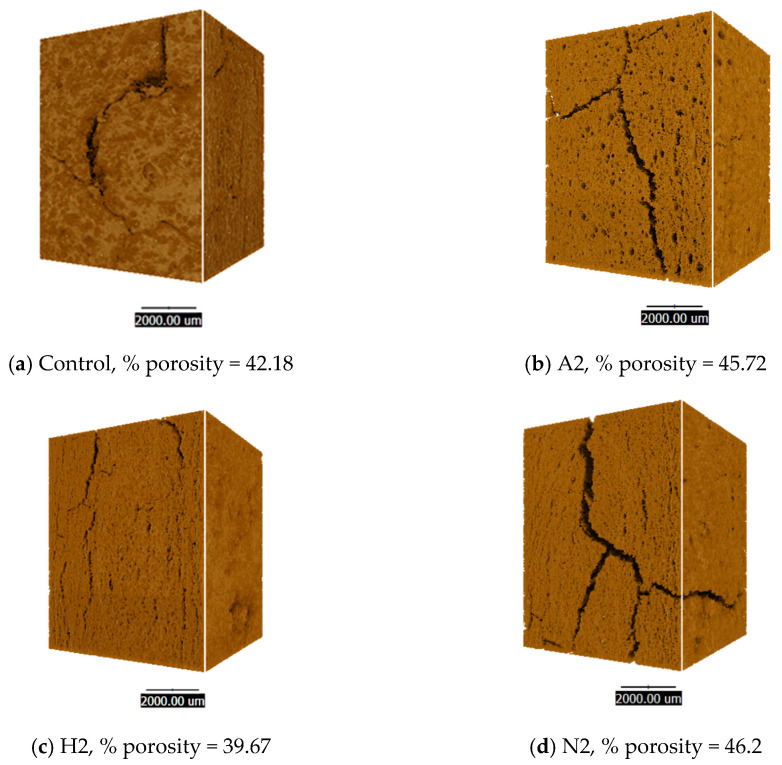
Quantitative analysis of porosity in high-moisture plant-based meat enriched with microalgae via Synchrotron Radiation X-ray Tomography Microscopy (SRXTM).

Figure 4 presents an analysis of porosity at 1.5% across the three strains of microalgae-containing HMMAs compared to the control sample. Synchrotron radiation X-ray tomography microscopy (SRXTM) resulted in a detailed three-dimensional representation that highlighted structural differences among the microalgae-based meat analogues, particularly in terms of their pore characteristics. Samples incorporating *H. pluvialis* demonstrated the smallest pore sizes, correlating with the lowest rehydration capacity. Smaller pore structures inherently restrict water absorption, thereby affecting the textural and functional properties of meat analogues.

### 3.7. Amino Acid Profiles and Digestibility

The amino acid composition of microalgae-containing HMMAs underscored the significance of these alternative proteins in promoting sustainability. Research has indicated that the integration of microalgae into HMMAs plays a crucial role in enhancing nutritional profiles. The essential amino acid profiles, as presented in Table 9, further emphasise the nutritional potential of these microalgae, highlighting their relevance in the development of sustainable food sources. Various experiments revealed the essential and non-essential amino acid profiles. No significant differences were found in amino acid compositions across the experiments, with the exception of cysteine, methionine, and proline. The incorporation of microalgae resulted in higher methionine levels than in the control group. The integration of microalgae into HMMAs enhanced the nutritional value and also contributed to the sustainability of food production. The ability of microalgae to thrive in diverse environments and their low resource requirements make them a promising ingredient for developing environmentally friendly and nutritionally rich protein sources.

**Figure 5:**
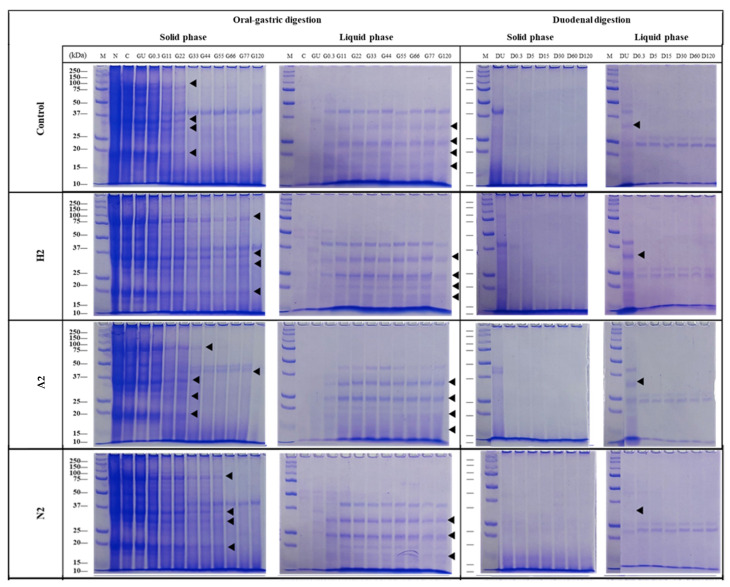
Protein patterns of soluble (liquid phase) and insoluble (solid phase) fractions after simulated oral-gastric digestion and oral-gastric-duodenal digestion at different time points of extruded products ((**A2**): *A. platensis*; (**H2**): *H. pluvialis*; (**N2**): *N. oculata*) and without microalgae (**control**). Note: The soluble and insoluble fractions at different time points were loaded in the same concentrations, taking into account the dilution factor between the digestion phases. M: protein marker; N: native protein; C: chew (oral phase); GU: gastric undigestion (without enzyme); DU: duodenal undigestion (without enzyme).

Protein digestion in HMMAs incorporating microalgae was evaluated. Figure 5 presents the electrophoretic patterns of high-moisture plant-based meat analogues with and without microalgae during in vitro digestion. This figure includes non-digested samples (native, N) as well as digesta collected at various time points. The control sample exhibited major protein bands ranging from 10 to 250 kDa. In the oral phase, most proteins in the sample were not soluble in the simulated salivary fluid, as indicated by the high band intensity in the solid phase. Protein digestion did not occur during this phase, as the band intensity in the chewed sample (C) was similar to the native sample (N). Proteins were more soluble in the simulated gastric fluid, particularly those with molecular weights of 35, 25, and below 20 kDa, possibly attributed to the pH differences between the simulated fluids. Gastric enzymes digested nearly all protein bands in the solid phase within 22 min (G22), resulting in lower molecular weight polypeptides. A decrease in band intensity was observed for polypeptides around 75, 35, 30, and 20 kDa, while an increase was seen for those below 15 kDa. By contrast, protein bands in the liquid phase remained visible until the end of the gastric phase. During the duodenal phase, the remaining polypeptides from the gastric phase were completely digested within 0.3 min, as evidenced by the disappearance of the corresponding bands. Polypeptides smaller than 10 kDa were detected. HMMAs incorporating microalgae underwent significant protein digestion, particularly in the gastric phase, leading to the formation of smaller polypeptides.

## 4. Discussion

Microalgae have garnered significant attention in recent years for their potential as a sustainable source of nutrition and bioactive compounds. Our findings revealed that *A. platensis* biomass had over 50% protein dry weight, making it a viable alternative protein source. By contrast, the *N. oculata* biomass protein content was more than 30% of its dry weight. The significantly elevated protein content, ranging from 55% to 70%, was markedly higher than that found in conventional protein sources such as beef (17% to 22%), chicken (19% to 22%), fish (19% to 22%), wheat (12% to 13%), or rice (8% to 10%) [53,54,55]. The protein content of alternative plant-based sources derived from soybeans typically ranges from 35% to 40%, while *A. platensis* has a higher protein content than soybeans [56]. *H. pluvialis* is a significant source of astaxanthin, a natural red pigment known for its antioxidant properties. *A. platensis*, *H. pluvialis*, and *N. oculata* have been recognised as rich sources of nutritional and bioactive pigment compounds. These properties highlight their potential application as functional ingredients in alternative food products, contributing to the enhancement of functional foods and health benefits. Plant-based meat analogues are food products formulated from plant-derived ingredients that aim to replicate the flavour, texture, and visual characteristics of traditional meat [57]. Their increasing popularity is driven by growing consumer demand for more sustainable and health-conscious protein sources, prompting the exploration of innovative alternative ingredients. Microalgae have emerged as an excellent option for incorporation as a functional ingredient in plant-based meat analogues. Our experiments involved two concentrations and three strains of microalgae, with results evaluated for physicochemical, nutritional, and techno-functional properties, followed by characterising the structure, amino acid profiles, and protein digestibility.

### 4.1. Athrospira-Enriched High-Moisture Meat Analogues

*Arthrospira* microalgae are added to extruded food items to improve their nutritional benefits. Previous studies of rice-soy crisps supplemented with 4% *Arthrospira* demonstrated an increase in protein content. These products can be successfully and effectively incorporated into school nutrition programs due to their shelf stability, palatability, economic viability, and capacity to fulfil children’s nutrient requirements [58]. The incorporation of *Arthrospira* sp. LEB 18 at an enrichment level of 2.6% into extruded snack formulations has been shown to enhance their physicochemical and structural properties. This innovative approach positions these snacks as a viable alternative health food option, contributing to increased protein content [59]. The incorporation of 2% bioactive peptides derived from *Arthrospira* sp. LEB 18 into extruded snack products significantly enhanced their antioxidant activity. This finding supports the potential for these snacks to offer health benefits through the integration of functional bioactive compounds [60]. A previous report on sustainable meat alternatives emphasised the potential of *Arthrospira* sp. incorporation in formulations ranging from 10% to 50% as a partial substitute for soy in extruded meat products. During the extrusion process, the moisture content fluctuates between 57% and 77% [61]. The incorporation of *Arthrospira* microalgae biomass into the extrusion of plant-based meat analogues has not been sufficiently explored in the existing literature. Various plant protein sources have been studied to determine the effects of adding *Arthrospira* on their nutritional value, but the characteristics and quality of meat substitutes remain largely unexamined, highlighting a significant research gap. Incorporating low concentrations of *A. platensis* into the HMMAs yielded a distinctive dark green colouration, while maintaining comparable moisture content and physicochemical properties to the control experiment. However, increasing the concentration of *Arthrospira* biomass amplifies the earthy flavour and intensifies the musty odour associated with algae [62]. This phenomenon was similar to the effect observed when elevating *Arthrospira* content from 5% to 15% in cookie formulations, which resulted in a more pronounced and undesirable musty-seawater or fishy-seawater odour [63]. When *H. pluvialis* microalgae biomass was incorporated under meat analogue conditions, the appearance resembled real meat. The total phenolic content (TPC) of sample A1 (1.09 mg GAE g^−1^) with *A. platensis* added was half that of the control (2.29 mg GAE g^−1^), due to two factors. First, the high protein content of *A. platensis* promoted protein–phenolic interactions during extrusion, making the phenolics non-extractable [64], and second, the extrusion conditions (60% moisture with temperature exceeding 120 °C) caused thermal degradation or structural changes in the phenolic compounds, reducing the amount of free TPC [65]. These results aligned with previous studies on cereal-legume extrudates, where extrusion led to decreased TPC due to complex formation and thermal loss [66], suggesting that the TPC did not fully represent the antioxidant potential when the phenolics were bound or modified.

### 4.2. Haematococcus-Enriched High-Moisture Meat Analogues

Our findings demonstrated that integrating microalgae biomass with plant-based ingredients effectively preserved colour stability during high-temperature food processing. This approach maintained the red natural pigment astaxanthin in *H. pluvialis* microalgae biomass. Previous studies highlighted that *H. pluvialis* microalgae serve as a rich source of the vibrant red natural colourant astaxanthin, offering beneficial antioxidant properties when incorporated into meat products [67]. Astaxanthin derived from the microalga *H. pluvialis* has been successfully incorporated into various meat products, resulting in significant enhancements in both colouration and functional properties. This bioactive carotenoid improves the visual appeal of the products and also confers additional health benefits through its antioxidant activity, thereby augmenting the nutritional profile and quality of meat products such as lamb from suckling animals, pork, and sausages [67,68,69]. Astaxanthin derived from *H. pluvialis* has been identified as the microalgal ingredient contributing to a red hue in textured plant protein, which is a key component in plant-based meat products [70]. Astaxanthin from *H. pluvialis* had higher thermostability within cooking temperatures [71]. Some microalgae-derived carotenoids are suitable for replicating the bright colouration of meat products after cooking that challenges plant-based meat analogues [72]. Moisture contents of the samples ranged between 55.51% and 60.83%. The H2 sample, incorporating *H. pluvialis*, had the lowest value, at 55.51%. This was below the typical target of 60% or higher for high-moisture meat analogues, but there were no evaporative losses during processing or transfer, because all the samples were handled under the same controlled conditions. The lowest moisture content in H2 was attributed to its denser matrix structure and smaller pore size observed in the microstructural analysis (Figure 4), which reduced water retention during extrusion.

### 4.3. Nannochlropsis-Enriched High-Moisture Meat Analogues

The biomass of *N. oculata* incorporated in the HMMA experiments had high textural integrity, along with chlorophyll and TPC. Previous research on *N. oceanica* biomass in plant-based foods showed that incorporating 30% *N. oceanica* into plant-based fishcake analogues significantly altered their textural properties. This change in texture influences the digestibility of proteins and lipids during in vitro digestion [73]. The incorporation of 2–3% *N. oceanica* into cookies and pasta ensures colour stability during storage, improves firmness values, and also serves as a valuable source of omega-3 fatty acids, thereby enhancing the nutritional profile of these products [74]. *N. oceanica* has been reported to enhance the levels of eicosapentaenoic acid (EPA) through the incorporation of pigments such as chlorophylls and carotenoids in various food products including pasta, tomato puree, dairy items, cookies, and bread [75]. Supplementation with *N. oceanica* biomass results in a fishy taste and odour in the final products [76].

### 4.4. Properties of Microalgae-Enriched High-Moisture Meat Analogues

The three microalgal strains tested in this research study can be utilised as food supplements, meat alternatives, natural food colourants, functional foods, and sustainable animal feed. Microalgae incorporated into meat analogues displayed colours originating from the natural pigmentation of each microalgal strain. The colour exhibited consistent patterns, with darker hues correlating with increased microalgae concentration. Following high-temperature extrusion during the meat analogue production process, the microalgae biomass was effectively integrated into the mixture, resulting in uniform and stable colouration across all meat analogue samples. However, challenges such as consumer acceptance, production costs, and product development need to be addressed to fully unlock the potential of microalgae. Educating consumers on the benefits and safety of microalgae, scaling up cultivation processes, and developing appealing microalgae-based food products are crucial steps for the future. Microalgae offer a promising and sustainable solution to meet the growing demand for nutritious and functional food ingredients. They are emerging as a highly sustainable source of protein, increasingly surpassing traditional meat and certain plant-based alternatives such as meat substitutes in terms of environmental and nutritional benefits [77]. There were significant improvements in the physicochemical, nutritional, textural, and functional properties of HMMAs incorporating microalgae compared to the control sample. A microalgae concentration of 1.5% was selected for subsequent detailed analysis, with particular emphasis on its water- and oil-holding capacities, with porosity evaluated via synchrotron radiation X-ray tomography microscopy.

Regarding protein and nutritional aspects, among the three species of microalgae added to high-moisture meat analogues, the low concentration of microalgae used resulted in no significant difference in protein levels between the experiments, with a protein content of over 50% in the HMMAs. The most commonly utilised plant-based protein sources for the production of fibrous structures include soybean, wheat gluten, and pea protein [78]. Previous research investigated alternative plant-based ingredients from various plant origins as protein sources for meat analogues produced utilising extrusion technology. The incorporation of rice and soy protein isolates into meat analogues resulted in enhanced nutritional value, reduced porosity, and decreased water absorption capacity [79]. Enzymatic modification of oat protein improved its functional properties, achieving high tensile strength and fibrous structure formation in high-moisture extrusion processes [80]. Lupin protein concentrate and isolate were developed for high-moisture extrusion [81], while HMMAs enriched with faba bean protein concentrate exhibited good firmness, elasticity, and fibrosity [82]. Adding different ingredients resulted in varied nutritional values and properties of the final high-moisture extrusion products. Nutritional values and quality are assessed based on protein content and balanced amino acid profiles to satisfy consumers’ dietary requirements [83]. Plant-based proteins frequently lack certain essential amino acids, thus rendering them nutritionally inadequate. Proteins derived from grains typically exhibit deficiencies in lysine, threonine, and tryptophan, whereas legumes usually contain insufficient levels of sulphur-containing amino acids such as cysteine and methionine. Various plant-derived protein sources can be combined to achieve a more balanced amino acid profile [84]. Our findings indicated that all the experiments involving the incorporation of microalgae influenced the amino acid composition in high-moisture meat analogues. High-moisture meat analogues (HMMAs) enriched with *A. platensis* and *N. oculata* demonstrated higher essential amino acid contents compared to HMMAs enriched with *H. pluvialis* and the control samples. Methionine was found in high concentrations in *A. platensis-* and *N. oculata*-enriched HMMAs. Low values of this amino acid can restrict product nutritional value [85]. The FAO/WHO/UNU recommends a daily intake of 13–15 mg per kg of body weight for the cysteine and methionine amino acids [86]. A high-moisture meat analogue enriched with microalgae, particularly *A. platensis* and *N. oculata*, was developed to enhance protein quality and meet essential amino acid requirements in human nutrition. In vitro protein digestion depends on several factors and varies by species; it is closely related to protein content, cell structure, and the food matrix. High-moisture extruded products containing *A. platensis* (A2), with a high protein content (more than 50% dry weight) and a relatively permeable cell wall, showed the highest gastric digestibility comparable to the control (C2), with most polypeptides digested within 22 min. This rapid digestion was consistent with the high solubility of *A. platensis* proteins and the absence of rigid cellulosic walls, as reported by Irvani et al. (2024)) [87]. By contrast, *H. pluvialis* (H2) and *N. oculata* (N2) had lower total protein contents and thicker, more recalcitrant cell walls composed of sporopollenin-like or algaenan polymers, which limit enzyme access to proteins during gastric digestion [88]. As a result, the digestion of most polypeptides occurred later—at 77 min for H2 and 55 min for N2—even though both species contained digestible proteins. These structural barriers delayed gastric breakdown but did not affect duodenal-phase digestibility, with all species achieving similar final protein hydrolysis. This highlighted the importance of both protein quantity and bioaccessibility when formulating microalgae-enriched HMMAs.

The incorporation of microalgae imparted distinct and species-specific pigmentation to the HMMAs, driven by differences in pigment composition: *A. platensis* contained phycocyanin and chlorophylls; *H. pluvialis* was rich in astaxanthin within the carotenoid group; and *N. oculata* provided chlorophylls and carotenoids. These pigments enhanced the visual appeal of the products by mimicking desirable meat-like or distinctive hues and also served as natural antioxidants. Astaxanthin, characterised by its long, conjugated polyene chain, effectively quenches singlet oxygen and scavenges peroxyl radicals, thereby protecting polyunsaturated fatty acids from oxidation [89]. Chlorophylls and carotenoids act as physical quenchers of reactive oxygen species (ROS) and stabilise lipid membranes against oxidative damage [90]. In this investigation, the pigments demonstrated high thermal stability during high-moisture extrusion, likely owing to their protection within the protein-lipid matrix, which decreased exposure to heat and oxygen, aligning with previous reports [28]. This dual functionality—offering stable natural colouration and enhancing oxidative stability—positioned microalgal pigments as multifunctional ingredients for the development of plant-based meats.

The results revealed that the texture and functionality of microalgae-enriched HMMAs were significantly affected by the biochemical composition and microstructural features of the extrudates. Rehydration capacity (RHC) improved in products supplemented with *A. platensis* and *N. oculata*, likely due to increased protein solubility and a more open pore network, which facilitated capillary water absorption—consistent with Bakhsh et al. [91]. By contrast, *H. pluvialis* exhibited lower RHC, attributable to a denser, smaller-pored structure observed through SRXTM imaging, corroborating previous studies indicating that compact matrices limit hydration [65]. The cooking yield also increased with the inclusion of *A. platensis* and *N. oculata*, suggesting that their hydrophilic proteins and soluble polysaccharides improved water and fat retention via hydrogen bonding and physical entrapment—as reported in other algae-fortified foods [92]. Water-holding capacity (WHC) showed no significant variation across species, with oil-holding capacity (OHC) was higher in *A. platensis* and *H. pluvialis*, due to the hydrophobic amino acids and lipid-binding pigments that interacted with lipids, and aligning with the emulsifying behaviours described by Teuling et al. [93]. Structural analysis supported these functional observations. *A. platensis* addition increased porosity, resulting in softer, less chewy products, *H. pluvialis* maintained a dense, rough surface, corresponding to lower RHC but elevated OHC, while *N. oculata* developed a honeycomb-like structure with increased hardness and cutting force—consistent with research suggesting that dense, reinforced networks confer greater mechanical strength [19]. Mechanistically, hydration relies on the availability of hydrophilic sites and pore openness, while oil retention involves hydrophobic domains and pigment–lipid complexes. Texture stability is maintained through protein–protein interactions and cell wall reinforcement [94,95]. These findings underscored how microalgae strain selection facilitated targeted modulation of hydration, lipid retention, and textural attributes in HMMAs. The functional effects were strain-specific and substantially affected by both the biochemical composition and microstructure of the microalgae.

The key parameters associated with microalgae incorporated into HMMAs are summarised in Table 10. Our findings indicated that various microalgae strains yielded distinct appearances and properties, which, in turn, influenced consumer acceptability. This highlighted the importance of strain selection in the development of appealing and functional meat alternatives. The integration of microalgae into HMMAs presents a promising avenue for developing sustainable, nutritious, and functional food products, which are crucial for advancing the field of plant-based meat alternatives and meeting the growing demand for sustainable protein sources.

## 5. Conclusions

Integrating microalgae into HMMAs is a significant advance in the development of sustainable and nutritionally adequate food sources. This study demonstrated that the addition of *Arthrospira platensis*, *Haematococcus pluvialis*, and *Nannochloropsis oculata* enhanced the physicochemical, functional, and nutritional attributes of HMMAs. Microalgal enrichment improved protein quality and augmented antioxidant capacity. The *H. pluvialis* specimens exhibited 1.8- and 2.0-fold increases in ABTS and FRAP antioxidant activities, respectively. *A. platensis* reduced hardness by 63%, whereas *N. oculata* increased the force required for cutting by 44%, thereby broadening consumer acceptance. *H. pluvialis* addition gave a product colour similar to traditional meat products. The incorporation of *A. platensis* and *N. oculata* enhanced the amino acid profiles, with increased levels of essential amino acids such as methionine. All the microalgae-enriched samples demonstrated high digestibility in vitro. Our findings underscored the potential of microalgal species as sustainable, health-promoting ingredients in meat substitutes, aligning with emerging consumer preferences for environmentally sustainable and health-conscious foods and opening promising avenues for novel protein sources. However, further investigations of the sensory attributes of flavour and aroma and large-scale production feasibility are required. Continued research in this domain is essential to address the dietary needs of a growing global population, while minimising the environmental impact associated with traditional animal agriculture.

## 6. Patents

The preparation methods of microalgae integrated into high moisture meat analogues and the production methods detailed in this research study are covered by a Thailand petty patent.

## Figures and Tables

**Table 1:** Biochemical composition (% dry basis) of microalgae biomass.

Composition	Protein	Lipid	Fibre	Ash	Carbohydrate
*A. platensis*	52.99 ± 3.78 ^c^	1.12 ± 0.07 ^a^	9.76 ± 0.08 ^c^	5.51 ± 0.18 ^b^	30.63 ± 3.45 ^a^
*H. pluvialis*	15.41 ± 1.51 ^a^	3.91 ± 0.06 ^b^	6.79 ± 0.28 ^b^	2.37 ± 0.21 ^a^	71.52 ± 1.93 ^c^
*N. oculata*	33.85 ± 0.25 ^b^	6.87 ± 0.10 ^c^	4.58 ± 0.66 ^a^	16.14 ± 0.01 ^c^	38.56 ± 0.30 ^b^

Data in the same column with different superscripts are significantly different (*p* < 0.05).

**Table 2:** Colour determination of microalgae incorporated into high-moisture extruded plant-based meat analogues.

Experiment	L*	a*	b*
Control	74.21 ± 0.36 ^f^	2.06 ± 0.12 ^c^	21.91 ± 0.42 ^cd^
A1	67.58 ± 0.99 ^e^	−0.71 ± 0.05 ^b^	18.76 ± 0.25 ^b^
A2	59.58 ± 0.37 ^b^	−3.34 ± 0.04 ^a^	17.03 ± 0.19 ^a^
H1	61.77 ± 0.77 ^c^	18.16 ± 0.61 ^d^	18.67 ± 0.33 ^b^
H2	43.76 ± 0.85 ^a^	26.73 ± 0.24 ^e^	16.76 ± 0.31 ^a^
N1	73.46 ± 0.52 ^f^	1.85 ± 0.10 ^c^	21.48 ± 0.29 ^c^
N2	66.02 ± 0.53 ^d^	1.80 ± 0.11 ^c^	22.13 ± 0.25 ^d^

Data in the same column with different superscripts are significantly different (*p* < 0.05).

**Table 3:** Values of pH, moisture content, water activity (a_w_), and textural integrity of microalgae incorporated into high-moisture extruded plant-based meat analogues.

Experiment	pH ^ns^	Moisture Content (% Wet Basis)	Water Activity (a_w_)	Textural Integrity (%)
Control	5.67 ± 0.05	62.38 ± 0.16 ^b^	0.89 ± 0.00 ^a^	28.72 ± 1.21 ^a^
A1	6.12 ± 0.06	64.82 ± 0.07 ^d^	0.89 ± 0.00 ^a^	30.01 ± 0.67 ^a^
A2	4.68 ± 2.19	65.58 ± 0.14 ^e^	0.89 ± 0.00 ^a^	29.47 ± 2.59 ^a^
H1	6.71 ± 0.10	63.17 ± 0.16 ^c^	0.89 ± 0.00 ^a^	29.60 ± 1.46 ^a^
H2	6.65 ± 0.01	55.51 ± 0.31 ^a^	0.89 ± 0.00 ^a^	28.47 ± 0.37 ^a^
N1	5.35 ± 0.01	62.22 ± 0.24 ^b^	0.90 ± 0.00 ^b^	37.15 ± 0.44 ^b^
N2	5.94 ± 0.01	67.51 ± 0.02 ^f^	0.91 ± 0.01 ^b^	39.16 ± 4.20 ^b^

Data in the same column with different superscripts are significantly different (*p* < 0.05). ns indicates non-significant differences among samples in the same column.

**Table 4:** Textural properties of high-moisture plant-based extruded meat analogues incorporated with microalgae.

Experiment	Cutting Force (N)	Hardness (N)	Chewiness (N)
Control	19.29 ± 1.87 ^a^	137.06 ± 8.53 ^c^	85.85 ± 4.38 ^c^
A1	26.72 ± 6.42 ^b^	128.35 ± 9.48 ^bc^	87.02 ± 6.47 ^c^
A2	24.43 ± 2.72 ^b^	86.68 ± 7.08 ^a^	60.35 ± 6.85 ^b^
H1	25.13 ± 1.69 ^b^	132.86 ± 8.48 ^c^	84.69 ± 5.99 ^c^
H2	38.91 ± 2.75 ^c^	120.08 ± 6.17 ^b^	75.48 ± 5.33 ^b^
N1	25.13 ± 2.74 ^b^	93.20 ± 7.27 ^a^	67.87 ± 5.12 ^ab^
N2	27.94 ± 5.87 ^b^	133.25 ± 9.57 ^c^	97.06 ± 7.40 ^d^

Data in the same column with different superscripts are significantly different (*p* < 0.05).

**Table 5:** Biochemical composition (% dry basis) of high-moisture extruded plant-based meat analogues incorporating microalgae.

Experiment	Protein ^ns^	Lipid	Fibre	Ash ^ns^	Carbohydrate ^ns^
Control	62.13 ± 0.35	0.18 ± 0.04 ^ab^	1.34 ± 0.17 ^abc^	4.08 ± 0.62	32.28 ± 0.14
A1	58.88 ± 4.69	0.59 ± 0.21 ^c^	1.49 ± 0.03 ^c^	4.29 ± 0.08	32.75 ± 4.79
A2	59.06 ± 1.70	0.18 ± 0.06 ^ab^	1.17 ± 0.11 ^ab^	4.31 ± 0.07	35.28 ± 1.80
H1	56.24 ± 0.43	0.36 ± 0.06 ^b^	1.13 ± 0.11 ^a^	4.27 ± 0.13	37.99 ± 0.51
H2	58.58 ± 1.97	0.10 ± 0.00 ^a^	1.39 ± 0.07 ^bc^	3.95 ± 0.19	35.98 ± 2.08
N1	61.05 ± 3.61	0.34 ± 0.02 ^b^	1.38 ± 0.03 ^bc^	3.98 ± 0.03	33.25 ± 3.54
N2	61.19 ± 0.37	0.28 ± 0.06 ^ab^	1.16 ± 0.04 ^ab^	3.93 ± 0.04	33.44 ± 0.43

Data in the same column with different superscripts are significantly different (*p* < 0.05). ns indicates non-significant differences among samples in the same column.

**Table 6:** Total chlorophyll and carotenoid contents of high-moisture extruded plant-based meat analogues incorporated with microalgae.

Experiment	Total Chlorophyll (mg/mg)	Total Carotenoids (µg/mg)
Control	ND	ND
A1	7.78 ± 0.38 ^ab^	4.11 ± 0.30 ^b^
A2	19.91 ± 1.05 ^d^	7.01 ± 0.28 ^c^
H1	6.52 ± 1.11 ^ab^	13.43 ± 0.99 ^d^
H2	9.45 ± 1.22 ^bc^	34.59 ± 1.80 ^e^
N1	5.60 ± 0.75 ^a^	2.01 ± 0.19 ^a^
N2	12.11 ± 2.31 ^c^	3.02 ± 0.36 ^ab^

ND: Not detected; data in the same column with different superscripts are significantly different (*p* < 0.05).

**Table 7:** Total phenolic contents and antioxidant properties of high-moisture extruded plant-based meat analogues incorporating microalgae.

Experiment	TPC	ABTS	FRAP
	(mg GAE/g)	(mg AAE/g)	(mg AAE/g)
Control	2.29 ± 0.02 ^b^	0.47 ± 0.00 ^a^	0.48 ± 0.04 ^a^
A1	1.09 ± 0.07 ^a^	0.65 ± 0.08 ^b^	0.87 ± 0.06 ^c^
A2	2.40 ± 0.19 ^bc^	0.68 ± 0.02 ^b^	0.81 ± 0.08 ^c^
H1	2.60 ± 0.07 ^d^	0.89 ± 0.08 ^c^	0.68 ± 0.01 ^b^
H2	2.23 ± 0.09 ^b^	0.84 ± 0.03 ^c^	0.98 ± 0.05 ^d^
N1	2.86 ± 0.13 ^e^	0.39 ± 0.01 ^a^	0.68 ± 0.01 ^b^
N2	2.56 ± 0.06 ^cd^	0.46 ± 0.06 ^a^	0.67 ± 0.04 ^b^

Data in the same column with different superscripts are significantly different (*p* < 0.05).

**Table 8:** Rehydration capacity and cooking yield of high-moisture extruded plant-based meat analogues incorporating microalgae.

Experiment	Rehydration Capacity (%)	Cooking Yield (%)
Control	22.38 ± 1.02 ^ab^	122.96 ± 0.11 ^bc^
A1	31.95 ± 0.40 ^d^	124.76 ± 0.09 ^d^
A2	28.20 ± 1.59 ^c^	125.58 ± 0.80 ^d^
H1	21.39 ± 0.71 ^ab^	122.52 ± 0.01 ^b^
H2	20.37 ± 0.29 ^a^	123.92 ± 0.00 ^cd^
N1	26.44 ± 1.64 ^c^	119.42 ± 0.82 ^a^
N2	24.99 ± 3.08 ^bc^	124.39 ± 0.20 ^a^

Data in the same column with different superscripts are significantly different (*p* < 0.05).

**Table 9:** Amino acid profiles (mg/100 g DW) of high-moisture extruded plant-based meat analogues incorporating microalgae.

Amino Acid	Control	A2	H2	N2
Aspartic acid *	4929.95 ± 181.57	5582.65 ± 453.68	4961.99 ± 466.57	5647.08 ± 498.53
Glutamic acid *	13,508.61 ± 490.81	14,862.75 ± 1406.91	13,418.75 ± 1319.40	15,323.39 ± 1223.74
Serine *	2705.74 ± 119.46	3088.08 ± 334.52	2748.53 ± 263.70	3123.60 ± 242.23
Histidine *	1010.50 ± 41.41	1140.27 ± 78.46	1010.99 ± 94.33	1133.40 ± 92.68
Glycine *	2248.64 ± 66.46	2614.27 ± 291.09	2329.51 ± 198.72	2570.41 ± 159.58
Threonine *	1183.85 ± 75.86	1312.34 ± 130.88	1170.69 ± 116.70	1361.08 ± 115.71
Arginine *	2257.15 ± 119.86	2429.65 ± 151.94	2341.98 ± 235.93	2659.62 ± 240.47
Alanine *	1896.25 ± 71.96	2176.26 ± 239.38	1920.97 ± 171.75	2189.91 ± 157.72
Tyrosine *	1351.16 ± 63.12	1523.61 ± 104.25	1339.85 ± 135.63	1564.79 ± 133.28
Cysteine	5311.31 ± 144.66 ^b^	4410.39 ± 275.73 ^a^	4274.20 ± 542.86 ^a^	5834.98 ± 57.36 ^b^
Valine *	777.03 ± 61.73	860.14 ± 77.87	773.98 ± 78.41	888.15 ± 76.66
Methionine	453.56 ± 24.00 ^a^	544.82 ± 31.18 ^ab^	476.14 ± 41.99 ^ab^	565.20 ± 45.44 ^b^
Phenylalanine *	2066.80 ± 103.87	2269.81 ± 201.21	2053.52 ± 202.70	2360.23 ± 191.64
Isoleucine *	646.84 ± 58.52	712.03 ± 63.99	642.14 ± 66.27	751.04 ± 69.15
Leucine *	2869.74 ± 160.77	3186.19 ± 281.31	2867.85 ± 283.23	3297.15 ± 272.44
Lysine *	1818.85 ± 70.72	1987.37 ± 150.05	1801.92 ± 173.20	2061.38 ± 153.50
Tryptophan *	190.42 ± 16.96	194.94 ± 16.98	196.77 ± 17.41	195.83 ± 3.26
Proline	2739.28 ± 34.15^d^	1188.55 ± 113.23 ^a^	1677.74 ± 40.57 ^b^	2255.17 ± 45.05 ^c^

Data within the same composition with different superscripts are significantly different (*p* < 0.05). * indicates non-significant differences among samples in the same row.

**Table 10:** Evaluation of the properties of microalgae-enhanced high moisture meat analogues.

Microalgae Species	Key Nutrients and Properties	Main Functional Effects	Optimal Inclusion Level in HMMA	Potential Limitations
*A. platensis*	High protein	Improved textural integrity, high protein digestibility, enhanced antioxidant capacity	1.5%	Earthy/musty odour at higher levels
*H. pluvialis*	High carotenoids (astaxanthin)	Stable red pigmentation after cooking, antioxidant activity	1.5%	Slower gastric digestion due to the cell wall structure
*N. oculata*	High lipid (EPA), carotenoids, chlorophyll	Stable green colour	1.5%	May impart a fishy odour, slower gastric digestibility

## Data Availability

The original contributions presented in this study are included in the article/Supplementary Material. Further inquiries can be directed to the corresponding author.

## Supplementary Materials

The following supporting information can be downloaded at: https://www.mdpi.com/article/10.3390/foods14162838/s1, Figure S1: Preliminary appearance of high-moisture meat analogue enriched with a maximum of 30% of *Arthrospira platensis*; Table S1: Barrel configuration of the twin-screw extruder (Clextral, Evolum 25) used in HMMA production..

## References

[1] Castro V., Oliveira R., Dias A.C.P. (2023). Microalgae and cyanobacteria as sources of bioactive compounds for cosmetic applications: A systematic review. Algal Res..

[2] Zhou L., Li K., Duan X., Hill D., Barrow C., Dunshea F., Martin G., Suleria H. (2022). Bioactive compounds in microalgae and their potential health benefits. Food Biosci..

[3] Eilam Y., Khattib H., Pintel N., Avni D. (2023). Microalgae—Sustainable Source for Alternative Proteins and Functional Ingredients Promoting Gut and Liver Health. Glob. Chall..

[4] Barkia I., Saari N., Manning S.R. (2019). Microalgae for high-value products towards human health and nutrition. Mar. Drugs.

[5] Ampofo J., Abbey L. (2022). Microalgae: Bioactive composition, health benefits, safety and prospects as potential high-value ingredients for the functional food industry. Foods.

[6] Martínez-Ruiz F.E., Andrade-Bustamante G., Holguín-Peña R.J., Renganathan P., Gaysina L.A., Sukhanova N.V., Puente E.O.R. (2025). Microalgae as Functional Food Ingredients: Nutritional Benefits, Challenges, and Regulatory Considerations for Safe Consumption. Biomass.

[7] Ayub A., Rahayu F., Khamidah A., Antarlina S.S., Iswari K., Supriyadi K., Mufidah E., Singh A., Chopra C., Wani A.K. (2025). Harnessing microalgae as a bioresource for nutraceuticals: Advancing bioactive compound exploration and shaping the future of health and functional food innovation. Discov. Appl. Sci..

[8] Kusmayadi A., Leong Y.K., Yen H.-W., Huang C.-Y., Chang J.-S. (2021). Microalgae as sustainable food and feed sources for animals and humans–biotechnological and environmental aspects. Chemosphere.

[9] Xu Y., Tong X., Lu Y., Lu Y., Wang X., Han J., Liu Z., Ding J., Diao C., Mumby W. (2024). Microalgal proteins: Unveiling sustainable alternatives to address the protein challenge. Int. J. Biol. Macromol..

[10] Lucas B.F., Brunner T.A. (2024). Attitudes and perceptions towards microalgae as an alternative food: A consumer segmentation in Switzerland. Algal Res..

[11] Panaite T.D., Cornescu G.M., Predescu N.C., Cismileanu A., Turcu R.P., Saracila M., Soica C. (2023). Microalgae (*Chlorella vulgaris* and *Spirulina platensis*) as a Protein Alternative and Their Effects on Productive Performances, Blood Parameters, Protein Digestibility, and Nutritional Value of Laying Hens’ Egg. Appl. Sci..

[12] Oslan S.N.H., Tan J.S., Oslan S.N., Matanjun P., Mokhtar R.A.M., Shapawi R., Huda N. (2021). Haematococcus pluvialis as a Potential Source of Astaxanthin with Diverse Applications in Industrial Sectors: Current Research and Future Directions. Molecules.

[13] Ha N.C., Tam L.T., Hien H.T.M., Thu N.T.H., Hong D.D., Thom L.T. (2024). Optimization of Culture Conditions for High Cell Productivity and Astaxanthin Accumulation in Vietnam’s Green Microalgae Haematococcus pluvialis HB and a Neuroprotective Activity of Its Astaxanthin. Bioengineering.

[14] Zanella L., Vianello F. (2020). Microalgae of the genus Nannochloropsis: Chemical composition and functional implications for human nutrition. J. Funct. Foods.

[15] Sathasivam R., Radhakrishnan R., Hashem A., Abd_Allah E.F. (2019). Microalgae metabolites: A rich source for food and medicine. Saudi J. Biol. Sci..

[16] Godfray H.C.J., Aveyard P., Garnett T., Hall J.W., Key T.J., Lorimer J., Pierrehumbert R.T., Scarborough P., Springmann M., Jebb S.A. (2018). Meat consumption, health, and the environment. Science.

[17] de Boer J., Schösler H., Aiking H. (2017). Towards a reduced meat diet: Mindset and motivation of young vegetarians, low, medium and high meat-eaters. Appetite.

[18] Tso R., Lim A.J., Forde C.G. (2021). A Critical Appraisal of the Evidence Supporting Consumer Motivations for Alternative Proteins. Foods.

[19] Dekkers B.L., Boom R.M., van der Goot A.J. (2018). Structuring processes for meat analogues. Trends Food Sci. Technol..

[20] Pietsch V.L., Bühler J.M., Karbstein H.P., Emin M.A. (2019). High moisture extrusion of soy protein concentrate: Influence of thermomechanical treatment on protein-protein interactions and rheological properties. J. Food Eng..

[21] Loveday S.M. (2020). Plant protein ingredients with food functionality potential. Nutr. Bull..

[22] Peng Y., Zhao D., Li M., Wen X., Ni Y. (2023). The Interactions of Soy Protein and Wheat Gluten for the Development of Meat-like Fibrous Structure. Molecules.

[23] Ning M., Ji Y., Zhang J., Pan H., Chen J. (2023). The Potential of Soluble Proteins in High-Moisture Soy Protein–Gluten Extrudates Preparation. Polymers.

[24] Naik H.R., Sekhon K.S. (2014). Influence of defatted soy flour addition on the quality and stability of pretzel type product. J. Food Sci. Technol..

[25] Pan-utai W., Iamtham S., Jacob-Lopes E., Queiroz M.I., Maroneze M.M., Zepka L.Q. (2023). Chapter 24—Techno-functional properties of microalgae in food products. Handbook of Food and Feed from Microalgae.

[26] Su M., Bastiaens L., Verspreet J., Hayes M. (2023). Applications of Microalgae in Foods, Pharma and Feeds and Their Use as Fertilizers and Biostimulants: Legislation and Regulatory Aspects for Consideration. Foods.

[27] Schreuders F.K.G., Schlangen M., Kyriakopoulou K., Boom R.M., van der Goot A.J. (2021). Texture methods for evaluating meat and meat analogue structures: A review. Food Control.

[28] Huang Z., Liu Y., An H., Kovacs Z., Abddollahi M., Sun Z., Zhang G., Li C. (2024). Utilizing Haematococcus pluvialis to simulate animal meat color in high-moisture meat analogues: Texture quality and color stability. Food Res. Int..

[29] Caporgno M.P., Böcker L., Müssner C., Stirnemann E., Haberkorn I., Adelmann H., Handschin S., Windhab E.J., Mathys A. (2020). Extruded meat analogues based on yellow, heterotrophically cultivated Auxenochlorella protothecoides microalgae. Innov. Food Sci. Emerg. Technol..

[30] Batista A.P., Niccolai A., Fradinho P., Fragoso S., Bursic I., Rodolfi L., Biondi N., Tredici M.R., Sousa I., Raymundo A. (2017). Microalgae biomass as an alternative ingredient in cookies: Sensory, physical and chemical properties, antioxidant activity and in vitro digestibility. Algal Res..

[31] Kyriakopoulou K., Dekkers B., van der Goot A.J., Galanakis C.M. (2019). Chapter 6—Plant-Based Meat Analogues. Sustainable Meat Production and Processing.

[32] Li K., Duan X., Zhou L., Hill D.R.A., Martin G.J.O., Suleria H.A.R. (2023). Bioaccessibility and bioactivities of phenolic compounds from microalgae during in vitro digestion and colonic fermentation. Food Funct..

[33] da Silva V.T., Mateus N., de Freitas V., Fernandes A. (2024). Plant-Based Meat Analogues: Exploring Proteins, Fibers and Polyphenolic Compounds as Functional Ingredients for Future Food Solutions. Foods.

[34] Kumar R., Hegde A.S., Sharma K., Parmar P., Srivatsan V. (2022). Microalgae as a sustainable source of edible proteins and bioactive peptides—Current trends and future prospects. Food Res. Int..

[35] Minekus M., Alminger M., Alvito P., Ballance S., Bohn T., Bourlieu C., Carrière F., Boutrou R., Corredig M., Dupont D. (2014). A standardised static in vitro digestion method suitable for food—An international consensus. Food Funct..

[36] García-Encinas J.P., Ruiz-Cruz S., Juárez J., Ornelas-Paz J.d.J., Del Toro-Sánchez C.L., Márquez-Ríos E. (2025). Proteins from Microalgae: Nutritional, Functional and Bioactive Properties. Foods.

[37] Pan-utai W., Iamtham S., Boonbumrung S., Mookdasanit J. (2022). Improvement in the Sequential Extraction of Phycobiliproteins from Arthrospira platensis Using Green Technologies. Life.

[38] Limsangouan N., Rodkwan N., Pengpinit W., Tumpanuvatr T., Pengpinit P., Paopun Y., Kantrong H. (2024). Physical property changes promoting shelf-life extension of soy protein-based high moisture meat analog under high pressure treatment. J. Food Sci. Technol..

[39] Kantrong H., Prasert W., Rodkwan N., Pengpinit W. (2022). Influence of Sacha inchi (*Plukenetia volubilis* L.) oil and extrusion process parameters on the quality of soya protein-based meat extender: An optimization approach. J. Food Process. Preserv..

[40] Charlie E.A., Angrainy H., Kantrong H. (2025). Exploring the use of rice bran and mung bean as soy substitutes in low-moisture extruded plant-based meat. Innov. Food Sci. Emerg. Technol..

[41] Horwitz W. (2010). Official methods of analysis of AOAC International. Volume I, agricultural chemicals, contaminants, drugs/edited by William Horwitz. AOAC Int..

[42] Lichtenthaler H.K., Wellburn A.R. (1983). Determinations of total carotenoids and chlorophylls a and b of leaf extracts in different solvents. Biochem. Soc. Trans..

[43] Pan-utai W., Boonpok S., Pornpukdeewattana S. (2021). Combination of mechanical and chemical extraction of astaxanthin from Haematococcus pluvialis and its properties of microencapsulation. Biocatal. Agric. Biotechnol..

[44] Park W.S., Kim H.-J., Li M., Lim D.H., Kim J., Kwak S.-S., Kang C.-M., Ferruzzi M.G., Ahn M.-J. (2018). Two Classes of Pigments, Carotenoids and C-Phycocyanin, in Spirulina Powder and Their Antioxidant Activities. Molecules.

[45] Campos Assumpção de Amarante M., Cavalcante Braga A.R., Sala L., Juliano Kalil S. (2020). Colour stability and antioxidant activity of C-phycocyanin-added ice creams after in vitro digestion. Food Res. Int..

[46] Renugadevi K., Valli Nachiyar C., Sowmiya P., Sunkar S. (2018). Antioxidant activity of phycocyanin pigment extracted from marine filamentous cyanobacteria Geitlerinema sp TRV57. Biocatal. Agric. Biotechnol..

[47] Gulzar S., Tagrida M., Martín-Belloso O., Soliva-Fortuny R. (2025). Optimizing high-moisture meat analogue textures through Artificial Intelligence: The effect of sorbitol in soy protein concentrate blends. LWT.

[48] Limaye A. Drishti, A Volume Exploration and Presentation Tool. Proceedings of the SPIE Optical Engineering + Applications.

[49] Dai Z., Wu Z., Jia S., Wu G. (2014). Analysis of amino acid composition in proteins of animal tissues and foods as pre-column o-phthaldialdehyde derivatives by HPLC with fluorescence detection. J. Chromatogr. B.

[50] Pantoa T., Baricevic-Jones I., Suwannaporn P., Kadowaki M., Kubota M., Roytrakul S., Mills E.N.C. (2020). Young rice protein as a new source of low allergenic plant-base protein. J. Cereal Sci..

[51] Pan-utai W., Pantoa T., Roytrakul S., Praiboon J., Kosawatpat P., Tamtin M., Thongdang B. (2023). Ultrasonic-Assisted Extraction and Antioxidant Potential of Valuable Protein from Ulva rigida Macroalgae. Life.

[52] Chini Zittelli G., Lauceri R., Faraloni C., Silva Benavides A.M., Torzillo G. (2023). Valuable pigments from microalgae: Phycobiliproteins, primary carotenoids, and fucoxanthin. Photochem. Photobiol. Sci..

[53] Koyande A.K., Chew K.W., Rambabu K., Tao Y., Chu D.-T., Show P.-L. (2019). Microalgae: A potential alternative to health supplementation for humans. Food Sci. Hum. Wellness.

[54] Dolganyuk V., Sukhikh S., Kalashnikova O., Ivanova S., Kashirskikh E., Prosekov A., Michaud P., Babich O. (2023). Food proteins: Potential resources. Sustainability.

[55] Lestingi A., Alagawany M., Di Cerbo A., Crescenzo G., Zizzadoro C. (2024). Spirulina (Arthrospira platensis) Used as Functional Feed Supplement or Alternative Protein Source: A Review of the Effects of Different Dietary Inclusion Levels on Production Performance, Health Status, and Meat Quality of Broiler Chickens. Life.

[56] Podgórska-Kryszczuk I. (2024). Spirulina—An Invaluable Source of Macro- and Micronutrients with Broad Biological Activity and Application Potential. Molecules.

[57] Benković M., Jurinjak Tušek A., Sokač Cvetnić T., Jurina T., Valinger D., Gajdoš Kljusurić J. (2023). An Overview of Ingredients Used for Plant-Based Meat Analogue Production and Their Influence on Structural and Textural Properties of the Final Product. Gels.

[58] Bashir S., Sharif M.K., Butt M.S., Rizvi S.S.H., Paraman I., Ejaz R. (2017). Preparation of Micronutrients Fortified Spirulina Supplemented Rice-Soy Crisps Processed Through Novel Supercritical Fluid Extrusion. J. Food Process. Preserv..

[59] Franco Lucas B., Morais M., Duarte Santos T., Costa J.A. (2017). Effect of Spirulina addition on the physicochemical and structural properties of extruded snacks. Food Sci. Technol..

[60] Silva P.C.d., Toledo T., Brião V., Bertolin T.E., Costa J.A.V. (2021). Development of extruded snacks enriched by bioactive peptides from microalga Spirulina sp. LEB 18. Food Biosci..

[61] Grahl S., Palanisamy M., Strack M., Meier-Dinkel L., Toepfl S., Mörlein D. (2018). Towards more sustainable meat alternatives: How technical parameters affect the sensory properties of extrusion products derived from soy and algae. J. Clean. Prod..

[62] Grahl S., Strack M., Mensching A., Mörlein D. (2020). Alternative protein sources in Western diets: Food product development and consumer acceptance of spirulina-filled pasta. Food Qual. Prefer..

[63] Nakib D., Ibrahim M., Mahmoud N., Abd E., Rahman E., Ghaly A., Preti R. (2019). Incorporation of Spirulina (Athrospira platensis) in Traditional Egyptian Cookies as a Source of Natural Bioactive Molecules and Functional Ingredients: Preparation and Sensory Evaluation of Nutrition Snack for School Children. Eur. J. Nutr. Food Saf..

[64] Šárka E., Sluková M., Henke S. (2021). Changes in Phenolics during Cooking Extrusion: A Review. Foods.

[65] Chiang J.H., Loveday S.M., Hardacre A.K., Parker M.E. (2019). Effects of soy protein to wheat gluten ratio on the physicochemical properties of extruded meat analogues. Food Struct..

[66] Ramos-Enríquez J.R., Ramírez-Wong B., Robles-Sánchez R.M., Robles-Zepeda R.E., González-Aguilar G.A., Gutiérrez-Dorado R. (2018). Effect of Extrusion Conditions and the Optimization of Phenolic Compound Content and Antioxidant Activity of Wheat Bran Using Response Surface Methodology. Plant Foods Hum Nutr.

[67] Pogorzelska E., Godziszewska J., Brodowska M., Wierzbicka A. (2018). Antioxidant potential of Haematococcus pluvialis extract rich in astaxanthin on colour and oxidative stability of raw ground pork meat during refrigerated storage. Meat Sci..

[68] Carballo D.E., Giráldez F.J., Andrés S., Caro I., Fernández-Gutiérrez M., Mateo J. (2019). Effects of dietary astaxanthin supplementation on the oxidative stability of meat from suckling lambs fed a commercial milk-replacer containing butylated hydroxytoluene. Meat Sci..

[69] Seo J.-K., Parvin R., Park J., Yang H.-S. (2021). Utilization of Astaxanthin as a Synthetic Antioxidant Replacement for Emulsified Sausages. Antioxidants.

[70] Liu M., Wang Y., Zhu L., Zhao X. (2023). Effects of Haematococcus pluvialis Addition on the Sensory Properties of Plant-Based Meat Analogues. Foods.

[71] Zhou Q., Xu J., Yang L., Gu C., Xue C. (2019). Thermal stability and oral absorbability of astaxanthin esters from Haematococcus pluvialis in Balb/c mice. J. Sci. Food Agric..

[72] Wu H., Sakai K., Zhang J., McClements D.J. (2024). Plant-based meat analogs: Color challenges and coloring agents. Food Nutr. Health.

[73] Zhao L., Khang H.M., Du J. (2024). Incorporation of microalgae (*Nannochloropsis oceanica*) into plant-based fishcake analogue: Physical property characterisation and in vitro digestion analysis. Food Hydrocoll..

[74] Srinivasan B., Kesavan R.k., Babu P.A.S., Sivarajan M., Sukumar M. (2014). Functional Foods Enriched with Marine Microalga Nannochloropsis oculata as a Source of ω-3 Fatty Acids. Food Technol. Biotechnol..

[75] Piyatilleke S., Thevarajah B., Nimarshana P.H.V., Ariyadasa T.U. (2024). Large-scale production of Nannochloropsis-derived EPA: Current status and perspectives via a biorefinery context. Food Bioprod. Process..

[76] Olsen M.L., Olsen K., Jensen P.E. (2024). Consumer acceptance of microalgae as a novel food—Where are we now? And how to get further. Physiol. Plant..

[77] Jafarzadeh S., Qazanfarzadeh Z., Majzoobi M., Sheiband S., Oladzadabbasabad N., Esmaeili Y., Barrow C.J., Timms W. (2024). Alternative proteins; A path to sustainable diets and environment. Curr. Res. Food Sci..

[78] Zhao Y.-R., Peng N., Li Y.-Q., Liang Y., Guo Z.-W., Wang C.-Y., Wang Z.-Y., Wang C., Ren X. (2024). Physicochemical properties, structural characteristics and protein digestibility of pea protein-wheat gluten composited meat analogues prepared via high-moisture extrusion. Food Hydrocoll..

[79] Lee J.-S., Oh H., Choi I., Yoon C.S., Han J. (2022). Physico-chemical characteristics of rice protein-based novel textured vegetable proteins as meat analogues produced by low-moisture extrusion cooking technology. LWT.

[80] Pöri P., Nisov A., Nordlund E. (2022). Enzymatic modification of oat protein concentrate with trans- and protein-glutaminase for increased fibrous structure formation during high-moisture extrusion processing. LWT.

[81] Palanisamy M., Franke K., Berger R.G., Heinz V., Töpfl S. (2019). High moisture extrusion of lupin protein: Influence of extrusion parameters on extruder responses and product properties. J. Sci. Food Agric..

[82] Saldanha do Carmo C., Knutsen S.H., Malizia G., Dessev T., Geny A., Zobel H., Myhrer K.S., Varela P., Sahlstrøm S. (2021). Meat analogues from a faba bean concentrate can be generated by high moisture extrusion. Future Foods.

[83] Yu J., Wang L., Zhang Z. (2023). Plant-based meat proteins: Processing, nutrition composition, and future prospects. Foods.

[84] Aghagholizadeh R., Rigi A.A. (2025). High-Moisture Extrusion in Plant-Based Meat: Challenges and Emerging Trends. J. Food Process Eng..

[85] Wang Y., Tibbetts S.M., McGinn P.J. (2021). Microalgae as Sources of High-Quality Protein for Human Food and Protein Supplements. Foods.

[86] Joint WHO/FAO/UNU Expert Consultation (2007). Protein and amino acid requirements in human nutrition. World Health Organization Technical Report Series.

[87] Irvani N., Leong S.Y., Carne A., Agyei D., Oey I. (2024). Impact of High-Speed Homogenisation Followed by pH Treatment of Arthrospira platensis on Protein Accessibility and In Vitro Protein Digestibility. Food Bioprocess Technol..

[88] Aydin S. (2016). Enhancement of microbial diversity and methane yield by bacterial bioaugmentation through the anaerobic digestion of Haematococcus pluvialis. Appl. Microbiol. Biotechnol..

[89] Ambati R.R., Phang S.M., Ravi S., Aswathanarayana R.G. (2014). Astaxanthin: Sources, extraction, stability, biological activities and its commercial applications--a review. Mar Drugs.

[90] García-Vaquero M., Brunton N., Lafarga T., Lafarga T., Acién G. (2021). Chapter 7—Microalgae as a source of pigments for food applications. Cultured Microalgae for the Food Industry.

[91] Bakhsh A., Park J., Baritugo K.A., Kim B., Sil Moon S., Rahman A., Park S. (2023). A holistic approach toward development of plant-based meat alternatives through incorporation of novel microalgae-based ingredients. Front. Nutr..

[92] Uribe-Wandurraga Z.N., Martínez-Sánchez I., Savall C., García-Segovia P., Martínez-Monzó J. (2021). Microalgae fortification of low-fat oil-in-water food emulsions: An evaluation of the physicochemical and rheological properties. J. Food Sci. Technol..

[93] Teuling E., Schrama J.W., Gruppen H., Wierenga P.A. (2019). Characterizing emulsion properties of microalgal and cyanobacterial protein isolates. Algal Res..

[94] Ubbink J., Muhialdin B.J. (2022). Protein physical state in meat analogue processing. Curr. Opin. Food Sci..

[95] De Angelis D., van der Goot A.J., Pasqualone A., Summo C. (2024). Advancements in texturization processes for the development of plant-based meat analogs: A review. Curr. Opin. Food Sci..

