# Supplementary material for: Microalgae-Enriched High-Moisture Meat Analogues: Improved Physicochemical, Functional, and Digestibility Properties
Source: Foods. 2025 Aug 16;14(16):2838. doi: 10.3390/foods14162838 (PMC12385683; doi:10.3390/foods14162838)
Supplement: Supplementary file 1 [file foods-14-02838-s001.zip › foods-3772705-supplementary.pdf]

# Microalgae-Enriched High-Moisture Meat Analogues: Improved Physicochemical, Functional and Digestibility Properties

**Wanida Pan-utai [1,*], Thidarat Pantoa [2], Waraporn Prasert [3], Janya Sangkhiaw [4], Catleya Rojviriya [5], Chalermluck Phoovasawat [5], Hataichanok Kantrong [3]**

[1] Department of Applied Microbiology, Institute of Food Research and Product Development, Kasetsart University, Bangkok, 10900, Thailand; ifrwdp@ku.ac.th
[2] Department of Food Chemistry and Physics, Institute of Food Research and Product Development, Kasetsart University, Bangkok, 10900, Thailand; ifrtrp@ku.ac.th
[3] Department of Food Processing and Preservation, Institute of Food Research and Product Development, Kasetsart University, Bangkok, 10900, Thailand; ifrhnk@ku.ac.th (H.K.); ifrwrpp@ku.ac.th (W.P.)
[4] Food Quality Assurance Services Center, Institute of Food Research and Product Development, Kasetsart University, Bangkok, 10900, Thailand; ifrjys@ku.ac.th
[5] Synchrotron Light Research Institute, 111 University Avenue, Suranaree, Mueang, Nakhon Ratchasima, 30000, Thailand; catleya@slri.or.th (C.R.); chalermluck@slri.or.th (C.P.)
* Correspondence: ifrwdp@ku.ac.th (W.P-u.)

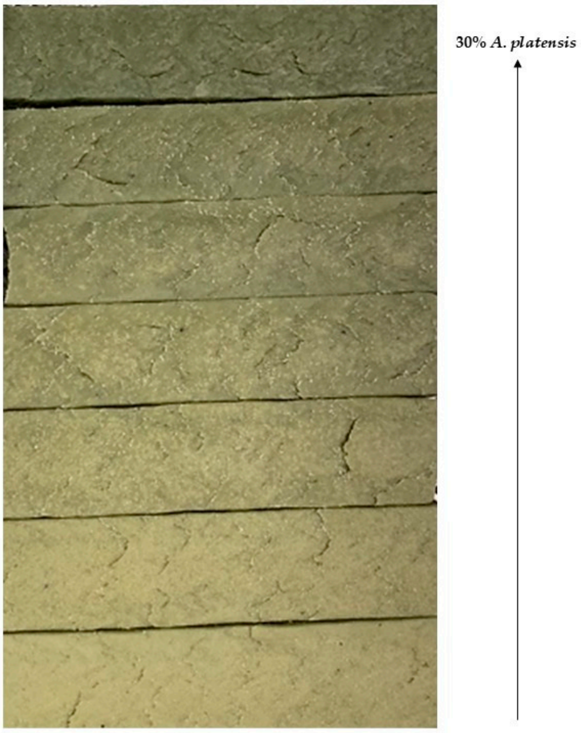


**Figure S1.** Preliminary appearance of high-moisture meat analogue enriched with a maximum of 30% of *Arthrospira platensis*.

**Table S1.** Barrel configuration of the twin-screw extruder (Clextral, Evolum 25) used in HMMA production.

| Barrel section (Zone) | Screw element type | Length (mm) | Function |
|---|---|---|---|
| Zone 1 | Conveying element | 100 | Initial feeding and transport |
| Zone 2 | Kneading block | 60 | Moderate mixing and hydration |
| Zone 3 | Conveying element | 100 | Material transition |
| Zone 4 | Kneading block | 60 | High shear and protein unfolding |
| Zone 5 | Reverse screw element | 30 | Pressure build-up and texturisation |
| Zone 6 | Conveying element (to die) | 90 | Forward flow and pressure maintenance |
